# The *Aspergillus nidulans* ATM Kinase Regulates Mitochondrial Function, Glucose Uptake and the Carbon Starvation Response

**DOI:** 10.1534/g3.113.008607

**Published:** 2013-11-05

**Authors:** Nadia Graciele Krohn, Neil Andrew Brown, Ana Cristina Colabardini, Thaila Reis, Marcela Savoldi, Taísa Magnani Dinamarco, Maria Helena S. Goldman, Gustavo Henrique Goldman

**Affiliations:** * Laboratório Nacional de Ciência e Tecnologia do Bioetanol–CTBE, Caixa Postal 6170, 13083-970 Campinas, São Paulo, Brazil; † Faculdade de Ciências Farmacêuticas de Ribeirão Preto, Universidade de São Paulo, São Paulo, Brazil; ‡ Faculdade de Filosofia, Ciências e Letras de Ribeirão Preto, Universidade de São Paulo, São Paulo, Brazil

**Keywords:** ATM kinase, glucose starvation, cell death, autophagy

## Abstract

Mitochondria supply cellular energy and also perform a role in the adaptation to metabolic stress. In mammals, the ataxia-telangiectasia mutated (ATM) kinase acts as a redox sensor controlling mitochondrial function. Subsequently, transcriptomic and genetic studies were utilized to elucidate the role played by a fungal ATM homolog during carbon starvation. In *Aspergillus nidulans*, AtmA was shown to control mitochondrial function and glucose uptake. Carbon starvation responses that are regulated by target of rapamycin (TOR) were shown to be AtmA-dependent, including autophagy and hydrolytic enzyme secretion. AtmA also regulated a p53-like transcription factor, XprG, inhibiting starvation-induced XprG-dependent protease secretion and cell death. Thus, AtmA possibly represents a direct or indirect link between mitochondrial stress, metabolism, and growth through the influence of TOR and XprG function. The coordination of cell growth and division with nutrient availability is crucial for all microorganisms to successfully proliferate in a heterogeneous environment. Mitochondria supply cellular energy but also perform a role in the adaptation to metabolic stress and the cross-talk between prosurvival and prodeath pathways. The present study of *Aspergillus nidulans* demonstrated that AtmA also controlled mitochondrial mass, function, and oxidative phosphorylation, which directly or indirectly influenced glucose uptake. Carbon starvation responses, including autophagy, shifting metabolism to the glyoxylate cycle, and the secretion of carbon scavenging enzymes were AtmA-dependent. Transcriptomic profiling of the carbon starvation response demonstrated how TOR signaling and the retrograde response, which signals mitochondrial dysfunction, were directly or indirectly influenced by AtmA. The AtmA kinase was also shown to influence a p53-like transcription factor, inhibiting starvation-induced XprG-dependent protease secretion and cell death. Therefore, in response to metabolic stress, AtmA appears to perform a role in the regulation of TOR signaling, involving the retrograde and SnfA pathways. Thus, AtmA may represent a link between mitochondrial function and cell cycle or growth, possibly through the influence of the TOR and XprG function.

The coordination of cell growth and division with nutrient availability is crucial for all microorganisms to successfully proliferate in a heterogeneous environment. The extracellular detection of nutrient availability and the intracellular sensing of energetic status induce a complex network of interlinked signal transduction pathways that subsequently regulate the appropriate cellular responses, coordinating metabolism and growth. The current understanding of how cell growth is controlled in response to nutrient availability is largely based on the study of mammalian systems in which several central protein kinases, including the AMPK (AMP-activated kinase) and TOR, were identified (Oldham and Hafen 2003; Fingar and Blenis 2004). *Saccharomyces cerevisiae* is a powerful model system for the study of nutrient sensing (Dechant and Peter 2008) and has provided a detailed understanding of nutrient availability signaling pathways. The mammalian kinases and signaling pathways implicated in the involvement in the control of cell growth are well-conserved in *S. cerevisiae* (Wilson and Roach 2002; de Virgilio and Loewith 2006; Busti *et al.* 2010; Rubio-Texeira *et al.* 2010; Santos *et al.* 2012).

The cAMP-dependent protein kinase A (PKA) and TOR pathways are essential for the promotion of *S. cerevisiae* cell growth and proliferation under nutrient-rich conditions. The cAMP–PKA pathway influences cell growth and sporulation via the activation of the Kss1/Fus3 mitogen-activated protein kinase (MAPK) cascades (Dechant and Peter 2008). The TOR kinases are activated by glucose and nitrogen sources (Barbet *et al.* 1996; Rolland *et al.* 2000) and during nutrient deprivation become inactive, resulting in a downregulation of cell growth and protein synthesis, while activating autophagy (Dechant and Peter 2008). Inactivation of either the cAMP–PKA pathway or the TOR pathway results in G1 arrest (Matsumoto *et al.* 1982; Barbet *et al.* 1986) and the activation of starvation responses (Gray *et al.* 2004), suggesting that PKA and TOR regulate cell growth by promoting G1 progression (Dechant and Peter 2008).

ATM is a serine/threonine protein kinase and a member of the phosphoinositide 3-kinase–related protein kinase family (Derheimer and Kastan 2010). Ataxia-telangiectasia is a rare autosomal-recessive disorder that causes progressive cerebellar ataxia, neurodegeneration, radio sensitivity, cell-cycle checkpoint defects, genome instability, and a predisposition for cancer (Boder and Sedgwick 1958; Kastan and Lim 2000; Lavin and Shiloh 1997). ATM plays a central role in coordinating the molecular events involved in DNA double-strand break signaling and repair (Langerak and Russell 2011; Stracker *et al.* 2013). Extensive evidence demonstrates how ATM is involved in the regulation of mitochondrial function, glucose homeostasis, serum starvation, and autophagy (Eaton *et al.* 2007; Halaby *et al.*, 2008; Alexander *et al.* 2010; Ching *et al.* 2010; Ditch and Paull 2012; Vazquez-Martin *et al.* 2011; Patel *et al.* 2011; Yang *et al.* 2011; Valentin-Vega and Kastan 2012; Valentin-Vega *et al.* 2012). In *A. nidulans*, the ATM homolog, AtmA, has an additional function in the regulation of polarized hyphal growth and the Δ*atmA* mutant has been shown to possess an accelerated rate of proliferation and increased nuclear kinetics (Malavazi *et al.* 2006, 2007). Interestingly, AtmA was recently also shown to be involved in the regulation of hydrolytic enzyme secretion (Brown *et al.* 2013). An interconnected network of activation between ATM, AMPK and TOR, in response to nutritional cues has been elucidated in mammals (Ditch and Paull 2012). Therefore, AtmA may also play a central role in the sensing of cellular energetic status.

Autophagy and apoptosis represent two distinct forms of programmed cell death (PCD) (Kourtis and Tavernarakis 2009). Autophagy forms part of a starvation response that is controlled by the highly conserved autophagy-related genes (ATGs). However, the function of autophagy is not restricted to nutrient recycling and is also involved in the removal of damaged proteins and/or organelles (Bursch *et al.* 2008). In mammals and *S. cerevisiae*, autophagic death can occur upon severe glucose deprivation and is regulated by the *atg1* kinase and TOR (Wullschleger *et al.* 2006; Levine and Kroemer 2008; Mizushima *et al.* 2008; Kourtis and Tavernarakis 2009). In *S. cerevisiae* and filamentous fungi, apoptotic-like cell death occurs during aging, reproduction, and after exposure to antifungal compounds (de Castro *et al.* 2011; Ramsdale 2008; Sharon *et al.* 2009). In *Aspergilli*, cell death is induced by carbon starvation (Nitsche *et al.* 2012; Szilágyi *et al.* 2013). Initially, autophagy is observed as an early starvation response (Nitsche *et al.* 2012; Szilágyi *et al.* 2013). Later, as a consequence of an apoptotic-like process and the degradation of cell wall biopolymers, hyphal fragmentation and autolysis occur (Emri *et al.* 2004, 2008).

The *A. nidulans* XprG transcription factor belongs to the p53 superfamily involved in gene regulation during starvation and subsequently influences cell death (Katz *et al.* 2006; Katz *et al.* 2009). The *xprG* gene was first identified in a genetic screen for mutants with elevated proteases secretion (Katz *et al.* 1996). The XprG homolog in *Neurospora crassa*, Vib-1, has also been shown to be involved in cell death during heterokaryon incompatibility (Dementhon *et al.* 2006). In mammalian cells, different residues of p53 protein are phosphorylated by ATM on exposure to distinct stimuli (Bensimon *et al.* 2011; Ditch and Paull 2012). In response to DNA damage activation and during the regulation of glucose homeostasis, ATM phosphorylates p53, resulting in its activation and an increase in protein stability (Shiloh 2006; Armata *et al.* 2010). In addition, ATM phosphorylates many other downstream protein kinases, with more than 700 proteins being shown to possess ATM phosphorylation motifs (Matsuoka *et al.* 2007). The reason for multiple ATM phosphorylation targets within each ATM-dependent pathway demonstrates how ATM functions to fine-tune various pathways influencing the same process.

The present study aimed to generate a better understanding of the involvement of AtmA in the regulation of mitochondrial function, glucose uptake, and autophagy during carbon starvation in *A. nidulans*. The loss of AtmA led to a reduction in glucose uptake, decreased respiratory capacity, and the elevated production of reactive oxygen species (ROS) representative of mitochondrial dysfunction. The role performed by AtmA during carbon starvation was investigated by transcriptional profiling. AtmA was shown to be involved in the control of extracellular hydrolase production and glucose uptake during carbon starvation. In addition, AtmA was shown to be involved in the regulation of XprG-dependent processes, including carbon starvation–induced protease secretion and cell death.

## Materials and Methods

### Strains and growth conditions

All *A. nidulans* strains used in this study are listed in Table 1. Complete media YG (2% w/v glucose, 0.5% w/v yeast extract, trace elements) and minimal medium MM (1% w/v glucose, nitrate salts, trace elements, pH 6.5) were used. The compositions of the trace elements, vitamins, and nitrate salts were described by Kafer (1977). According to strain auxotrophy, the media was supplemented with 1.2 g/liter of uracil and uridine (for *pyrG* auxotrophs), 1 g/liter of aminobenzoate (for *pabaA* auxotrophs), or 0.5 g/liter of pyridoxine HCl (for *pyroA* auxotrophs). Solid derivatives of YG and MM were prepared via the addition of agar (2% w/v). Before starvation, 1×10^7^ conidia were incubated in 50 ml liquid MM at 37° in a rotatory shaker (180 rpm) for 24 hr. Subsequently, mycelia were washed with distilled water and then incubated in glucose-free MM at 37° for 12, 24, 48, 96, or 192 hr.

**Table 1:** *Aspergillus nidulans* strains used in this study

Strain	Genotype*^a^*	Reference
MK85	*biA1*; *cho1*; *xprG1*; *niiA4*	Katz *et al.* (1996)
MK414	*pabaA1*; *yA2*; *argB2*; *xprGΔ1(xprG*:: *argB)*	Katz *et al.* (2006)
GFPatg8	*pyrG89*; *argB2*; *argB*::*ΔnkuA*; *pyroA4 atgH*::*GFP pyrG*	Mark Marten’s laboratory
*alcA*::*xprG*	*cho1*; *pyroA4 pyrG89*; *argB2*; *argB*::*ΔnkuA*; *aclA xprG*::*pyrG89*	This study
ACS16 *alcA*::*xprG*	*wA3*; *ΔatmA*; *alcA xprG*	This study
R21	*yA1 pabaA1*	Fantes and Roberts (1973)
ACS16	*pyrG89*; *pyroA1*; *wA3*; *ΔatmA*::*pyrG(FOA)*	Malavazi *et al.* (2006)
MK85ACS16	*wA3*; *xprG1*; *ΔatmA*	This study
MK414ACS16	*wA3*; *pyrG89*; *xprGΔ1*, *ΔatmA*	This study
ACS16 GFPatg8	*wa3*; *ΔatmA*; *atgH*::*GFP*	This study
*Δatg1*	*pyrG89*; *wA3*; *argB2*; *ΔnkuA^ku70^*::*argB pyroA4*; *sE15 nirA14 chaA1fwA1*; *Δatg1*::*pyrG89*	FGSC*^b^*
*Δatg1* atg8::GFP	*cho1*; *Δatg1*; *atgH*::*GFP*	This study

a For the meanings of gene symbols, see Clutterbuck (1993).

b Fungal Genetics Stock Center (http://www.fgsc.net).

### Oxygen uptake measurements

Wild-type and *ΔatmA* germlings were obtained by incubating a total of 1×10^8^ conidia in 50 ml MM for 16 hr at 37° (250 rpm). The germlings were harvested by centrifugation and washed twice with cold distilled water. Precipitated germlings were incubated for 5 hr (90 rpm) in a standard *A. nidulans* protoplasting solution (Novozyme 234 from Novo Nordisk was used as a lytic enzyme) (Osmani *et al.* 1987) at 30° to partially lysis the cell wall to facilitate respiratory substrate and inhibitors entry. After incubation, germlings were washed three times with 0.7 mM sorbitol, 10 mM HEPES–KOH, pH 7.2, and were subsequently kept on ice. Oxygen uptake was measured with a Clark-type electrode fitted to a Gilson oxygraph (Gilson Medical Electronics, Middleton, WI) in 1.8 ml of medium containing 0.7 mM sorbitol, 10 mM HEPES–KOH, pH 7.2, 5 mM MgCl_2_, and 0.5 mM EGTA at 30° (Dinamarco *et al.* 2010). The initial solubility of oxygen in the reaction buffer was considered to be 445 ng atoms of oxygen per ml. Further additions are represented in Table 2.

**Table 2:** Oxygen consumption in the wild-type and *ΔatmA* mutant strain

Treatments*^a^*	Wild-type	*ΔatmA*
Respiration medium	43.90 ± 3.2	21.40 ± 4.06
Antimycin A*^b^*	30.10 ± 1.2	14.03 ± 3.45
SHAM*^c^*	3.25 ± 0.3	2.10 ± 1.52
TMPD/Ascorbate*^d^*	28.66 ± 0.9	11.85 ± 1.50
KCN*^e^*	2.20 ± 0.65	3.80 ± 0.80

a Mean ± SD of three independent experiments. The values represent the rate of oxygen uptake and were expressed as ng atoms O/min/3 ×10^7^ cells.ml^−1^.

b 0.5 µg Antimycin A.

c 2.5 mM SHAM (salicylhydroxamic acid).

d 150 µM TMPD (N.N.N.N-tetramethyl-p-phenylenediamine) and 0.5 mM ascorbate (ASC).

e 1.0 mM KCN.

### Mitochondrial mass measurements

Conidia from wild-type and *ΔatmA* strains (1×10^8^ ml^−1^) were incubated in 50 ml liquid MM at 37° on a reciprocal shaker for 6 hr. Subsequently, conidia were washed with phosphate-buffered saline (PBS; 140 mM NaCl, 2 mM KCl, 10 mM NaHPO_4_, 1.8 mM KH_2_PO_4_, pH 7.4) and incubated with 8 nM MitoTracker Green FM (Invitrogen) or 5 nM Nonyl Acridine Orange (NAO; Invitrogen) diluted in PBS plus 5% fetal bovine serum (FBS) for 10 min at 37°. Stained conidia were washed with PBS, resuspended in PBS plus 5% FBS, and then analyzed by flow cytometry. Propidium iodide (0.5 µM) staining of the nucleus was utilized to exclude dead cells. Flow cytometry was analyzed by guava Easycity 8 HH (Millipore) using 10,000 acquisitions for each analysis.

For cytochrome *c* measurements, 2×10^8^ conidia/ml of wild-type and *ΔatmA* strains were inoculated in MM for 16 hr at 37°. After this period, the cultures were filtered and washed with 100 ml H_2_O, immediately frozen in liquid nitrogen and macerated. For every 0.1 g of dry weight, 1.3 ml HB buffer (150 mM NaCl, 30 mM KCl, 10 mM Na_2_HPO_4_, pH 7.0) was added. The suspension was centrifuged at 21,000 rpm at 4° for 30 min. The supernatant was removed and centrifuged again for 10 min under the same conditions. After determining protein concentration, samples were prepared for SDS-PAGE by the addition of 1× sample buffer (62.5 mM Tris-HCl pH 6.8, 2% SDS, 10% glycerol, 5% β-mercaptoethanol, and 5% bromophenol blue) and heating at 100° for 3 min. Thirty micrograms of total protein from each sample were loaded into each lane of a 15% SDS-PAGE gel. After separation of the proteins, the gel was blotted onto a pure nitrocellulose membrane (0.2 μm; Bio-Rad) and after being blocked in 5% dried milk in TBS-T buffer (10 mM Tris-HCl, 150 mM NaCl, pH 8.0, and 0.05% Tween 20), the membrane was probed with the rabbit anti-cyc1 antibody (CNAT against native cytochrome *c* from yeast; Sigma) at a 1:200 dilution in TBS-T buffer for 1 hr at room temperature. The membrane was washed four times with TBS-T buffer for 5 min and then incubated with a 1:5000 dilution of goat anti-rabbit IgG peroxidase-labeled (KPL) antibody for 1 hr. After being washed, the blot was developed by use of the SuperSignal Ultra chemiluminescence detection system (Pierce) and recorded by the use of Hyperfilm ECL (Amersham Biosciences).

### Glucose uptake assay

Glucose uptake rates were measured by assessing the incorporation of D-[U-^14^C] glucose [250–360 mCi (9.25–13.33 GBq)/mmol; Perkin Elmer Life Sciences] into germinating conidia. Briefly, 1.2×10^9^ conidia was inoculated into 300 ml MM containing 2% glucose (w/v) as a carbon source, and incubated for 6 hr at 37° in an orbital shaker (180 rpm). Germinating conidia were harvest by centrifugation (3000 rpm) for 5 min and washed four times with ice-cold MM without a carbon source to eliminate traces of glucose. Washed conidia were gently resuspended. For the glucose transport analysis, aliquots of 250 µl (of 2.5×10^7^ germinating conidia) containing D-glucose (0.1–20 mM) were dispensed into 2-ml tubes plus 1 µl radiolabeled glucose (0.2 µCi). Incubation times ranged from 30 to 180 sec at 37° with shaking. To quench the reaction, 1 ml ice-cold MM without carbon was added. The germinating conidia were washed twice with 1 ml ice-cold MM without carbon and transferred to 8 ml ScintiSafe Econo1 scintillation liquid (Fisher Scientific). The D-[U-^14^C] glucose taken-up by cells was measured using Tri-Carb 2100TR Liquid Scintillation Counter.

### ROS detection

Intracellular ROS levels were monitored with the oxidant-sensitive probe 5-(and 6)-chloromethyl-2′,7′-dichlorofluorescin diacetate CM-H_2_DCFDA (Invitrogen). Liquid YUU medium (50 ml) was inoculated with 1×10^7^ conidia and incubated on a rotatory shaker (180 rpm) for 6 hr at 37°. The nonstarved cultures were centrifuged at 4000 rpm and the pellet was resuspended in 2 ml YUU. The starved culture was centrifuged at 4000 rpm, the pellets were washed in MM without any carbon source, centrifuged again, resuspended in MM without a carbon source and incubated for another 1 hr to starve the culture. The starved cultures were centrifuged at 4000 rpm and the pellet was resuspended in 2 ml MM without a carbon source. After starvation, the ROS assay was prepared from 20 µl of either culture (starved or nonstarved) plus 180 µl fresh YUU or MM without a carbon source and 2.9 µg/ml CM-H_2_DCFDA in a 96-well imaging plates (BD Falcon). The assay plate was incubated at 37° for 30 min under shaking. The arbitrary fluorescence units (AFUs) were measured at 503 nm of excitation and 529 nm of emission in the fluorimeter Synergy (Biotek) using the Gen5 software.

### RNA isolation, cDNA synthesis and quantitative PCRs

Mycelia were harvested by filtration, washed with sterile water, immediately frozen in liquid nitrogen and ground into a powder while frozen. Total RNA was extracted with Trizol (Life Technologies) and RNAse-free DNAse treated as described by Semighini *et al.* (2002) and then purified using the RNeasy Plant Mini Kit (Qiagen). RNA integrity was confirmed on a bioanalyzer (Agilent). The synthesis of cDNA was performed with approximately 10 µg of RNA, 150 pmol oligo (d)T, and 200 units Superscript II (Invitrogen) according to manufacturer’s instructions. Quantitative PCRs were performed as previously described by Semighini *et al*. 2002. Primers used are list in Table S1. Expression of the tubulin gene *TubC* (AN6838) was used as an endogenous control for *XprG* normalisation.

### Microarray slide construction and gene expression methods

*A. nidulans* genes transcriptionally regulated after exposure to carbon starvation for 12 hr and 24 hr were identified using Agilent custom-designed oligonucleotides arrays (4× 45K microarray). The two strains used in this study were the wild-type and *ΔatmA*. The 0 hr starvation time point (growth in glucose 2% for 24 hr) was used as a hybridization reference for each strain. To construct the microarray slides, the Agilent E-array software tool was used (available at https://earray.chem.agilent.com/earray/). Briefly, we uploaded gene sequences representing the whole *A. nidulans* A4 gene sequences. The ORF number was carefully validated by comparing the sequences deposited in three databanks [CADRE (The Central *Aspergillus* Resource), AspGD (*Aspergillus* Genome Database), and BROAD Institute] aiming to identify and validate the sequences for probe design, which resulted in 11,251 ORFs being submitted to Agilent E-array. Based on some quality parameter implemented in Aglilent E-array (such as sequences with high scores for cross-hybridization potential throughout the genome and sequences that no appropriate regions could be found as targets to be represented in the slides), 11,143 probes were designed from the uploaded sequence of the A4 strain. These probes were represented three to four times in the microarray slides and the annotation based on [1, 2] was used to generate the annotation file used in the analysis. Therefore, the microarray slides comprised 45,220 features including 1417 Agilent internal controls and 800 internal controls that represented 80 randomly chosen *A. nidulans* ORFs (each 10-times replicated).

The gene expression analysis used in this work was performed using custom-designed oligonucleotides slides (4× 44K microarray) from Agilent Technologies based on *A. nidulans* genome annotation publicly available. After RNA isolation and purification, the samples were labeled with Cy-3 or Cy-5-dUTP using the two-color microarray-based gene expression analysis (Quick Amp Labeling Kit; Agilent Technologies) following the manufacturer’s protocol. Initially, 200 ng total RNA was incubated with the Agilent RNA Spike-In control probes (RNA Spike A or B mix). Before labeling, synthesis of cRNA was performed by incubating the samples with 1.2 µl T7 promoter primer and nuclease-free water in an appropriate volume. The template and primer were denatured by incubating the reaction at 65° in a circulating water bath for 10 min, and after the reactions were placed on ice for 5 min. cRNA Master Mix (4 µl 5× First Strand Buffer, 2 µL 0.1 M DTT, 1 µl 10 mM dNTP mix, 1 µl MMLV-RT, and 0.5 µl RNaseOut) were added to the samples, and the mixture was incubated at 40° in a circulating water bath for 2 hr. After, the samples were moved to a 65° circulating water bath and incubated for 15 min. cRNA amplification and labeling were performed by adding the Agilent Transcription Master Mix (20 µl 4× transcription buffer, 6 µl 0.1 M DTT, 8 µl NTP mix, 6.4 µl 50% PEG, 0.5 µL RNase OUT, 0.6 µl inorganic pyrophosphatase, 0.8 µl T7 RNA polymerase, 2.4 µl Cyanine 3-CTP to control samples, or cyanine 5-CTP to treated samples, and 15.3 µl nuclease-free water) to the samples and incubating the mixtures in a circulating water bath at 40° for 2 hr. The labeled cRNA was purified using RNeasy Mini Kit (Qiagen) and then quantified in the NanoDrop 2000 Thermo Scientific (Uniscience).

For the hybridization, 825 ng of each labeled cRNA was mixed with Agilent Fragmentation Mix (11 µl 10× blocking agent, 2.2 µl 25× fragmentation buffer, and nuclease-free water to bring the volume to 52.8 µl) and incubated at 60° for exactly 30 min to fragment RNA. The fragmentation was interrupted by adding 55 µl of 2× GE Hybridization Buffer HI-RPM. Finally, 100 µL sample was placed onto the microarray slide, which was mounted in the Agilent Microarray Hybridization Chamber Kit. The hybridization was performed in an Agilent G2545A Hybridization Oven set to 65° for 17 hr. After, microarray slides were washed according to Agilent’s instructions and scanned using GenePix 4000B microarray scanner (Molecular Devices).

### Gene expression analysis

The extraction of data from TIFF images generated through scanning of microarray slides was performed by using Agilent Feature Extraction (FE) software version 9.5.3.1 (Agilent Technologies) using Linear Lowess algorithm to obtain background subtracted and normalized intensity values. The dye-normalized values generated in the FE data files were uploaded into the software Express Converter (version 2.1; TM4 platform available at http://www.tm4.org/utilities.html), which converts the Agilent file format to a mev (multi-experiment viewer) file format compatible with the TM4 software for microarray analysis (available at http://www.tm4.org/). The mev files were then uploaded in the MIDAS software (TM4 platform), where the resulting data were averaged from replicated genes on each array from three biological replicates of each treatment. The generated mev files were finally analyzed by using TIGR MeV (TM4 platform, multi-experiment viewer; available at http://www.tigr.org/software/microarray.shtml), whereby differentially expressed genes were statistically identified using one-class *t* test (*P* > 0.01). Significantly different genes were those whose mean log_2_ expression ratios was statistically different from 0, which indicates the absence of gene modulation. The full dataset was deposited in the Gene Expression Omnibus (GEO) from the National Center of Biotechnology Information (NCBI) with the number http://www.ncbi.nlm.nih.gov/geo/query/acc.cgi?acc=GSE42732.

### Staining and microscopy

Sterile coverslips were overlaid with 5 ml liquid YUU medium containing approximately 1×10^6^ conidia and incubated at 25° for 12 hr before starvation. Starvation was induced by replacing YUU with MM without carbon and incubating at 25° for different time periods. Germlings starved of carbon for 12-hr and 24-hr were fixed (3.7% formaldehyde; 50 mM Pipes, pH 6.7; 25 mM EGTA, pH 7.8; 5% dimethyl sulfoxide) for 15 min and washed three times for 5 min in PEM (50 mM Pipes, 25 mM EGTA, 5 mM MgS0_4_). Cell walls were partially permeabilized with a digestion solution [50 mg/ml lysing enzymes (Lallzyme Mmx; Lallemand), 6 mg/ml BSA (Sigma) solubilized in PEM] for 1 hr at 30°. After cell wall permeabilization, germlings were washed for 10 min in PEM and then PEM plus BSA (1% w/v). Finally, the germlings were stained with TUNEL (In Situ Cell Death Detection Kit; Roche Diagnostics) for 1 hr at 37°, with 100 ng/ml Hoescht 33258 (Molecular Probes) for 2 min and subsequently washed three times with PEM plus BSA. Propidium iodide (3.75 µg/ml) and Hoescht double staining was performed at room temperature for 2 min and then washed three times for 5 min with PBS. The samples were examined using a Zeiss epifluorescence microscope with excitations of 359, 498, 536, and 563 nm and emissions of 461, 516, 617, and 582 nm for Hoescht, GFP, PI, and TUNEL, respectively. The phase contrast bright field and fluorescent images were captured with AxioCam camera (Carl Zeiss) and processed using the AxioVision software version 3.1.

To determine mitochondrial mass, the strains were grown on coverslips overlaid with MM containing 1% glucose. MitoTracker Green FM (8 nM) and NAO (5 nM) staining were performed at 37° for 10 min. The germlings were examined using a Zeiss epifluorescence microscope with MitoTracker Green FM excitations of 470/20 and emissions of 525/50 and NAO excitations of 572/25 nm and emissions of 626/62 nm. The phase contrast bright field and fluorescent images were captured with AxioCam camera (Carl Zeiss) and processed using the AxioVision software version 3.1.

### Construction of the alcA::xprG mutant

The strategy to overexpress the *xprG* gene was based on the replacement of its promoter with the *alcA* promoter (Flipphi *et al.* 2002). First, a 1087-bp DNA fragment from the 5′ unstranslated region (UTR) from the *xprG* gene was PCR-amplified from *A. nidulans* wild-type genomic DNA by using the 5′ UTR XprG pRS426 forward and 5′ UTR XprG pyrG reverse primers. Then, a 1900-bp DNA fragment of the *A. fumigatus pyrG* marker gene was PCR-amplified from the pCDA21 vector by using the pyrG alcA forward and pyrG alcA reverse primers. Subsequently, the *alcA* minimal promoter (307-bp) was PCR-amplified from the pMCB17apx vector with pyrG alcA forward and pMCB17 alcA reverse primers. Finally, to allow homologous integration at the 3′-end, a 1159-bp DNA fragment of the *xprG* ORF was PCR-amplified with the primers alcA orf XprG and pRS426 orf XprG reverse. Because each DNA fragment has approximately 20-bp overlapping sequences, it was possible to assemble them by mixing equal proportions of the four fragments described and PCR-amplifying them by using the external primers 5′ UTR XprG pRS426 forward and pRS426 orf XprG reverse. The PCR-mediated assembled DNA fragment was transformed into the *A. nidulans* TNO2A3 (*ΔnkuA*) strain (Nayak *et al.* 2006) according to standard protocols (Osmani *et al.* 1987). Primer sequences are described in the Supporting Information, Table S1.

### Protease activity

Two assays were used to test protease activity. For the semi-quantitative assay, the different strains were grown for 48 hr at 37° on solid MM plus 1% milk powder as a sole carbon source. Plates containing the *alcA*::*xprG* and *ΔatmA alcA*::*xprG* mutant strains were supplemented with 10 mM cyclopentanone. The formation of a halo of protein degradation, representative of protease secretion, was evaluated and the clearance index calculated (diameter of clearance zone / diameter of colony). The quantitative method used the fluorescence resonance energy transfer (FRET) peptide library Abz GXXXXXQ-EDDnp (Oliveira *et al.* 2012); 100 µl supernatant from 48-hr carbon-starved cultures was mixed with 100 µl buffer (sodium acetate 100 mM, pH 4.5; sodium phosphate 250 mM, pH 7.0; or tris-HCl 100 mM, pH 8.5) and 1 µl of the Abz GXXXXXQ-EDDnp peptide library (1 mg/ml of DMSO) was added. The FRET peptide library is composed of Abz (or MCA)-GXXXXXQ-EDDnp and Abz (or MCA)-GXXZXXQ-EDDnp, where X stands for an equimolar mixture of all amino acids, the Z position is fixed with one of the proteinogenic amino acids (cysteine was excluded), Abz (ortho-aminobenzoic acid) is the fluorescence donor, and Q-EDDnp (glutamine-[N-(2,4-dinitrophenyl)-ethylenediamine]) is the fluorescence acceptor (Oliveira *et al.* 2012). Reactions were performed within 96-well imaging plates (BD Falcon) at 30° for 30 min under shaking. The AFUs were measured in a fluorimeter Synergy (Biotek) using the Gen5 software (excitation 320 nm, emission 420 nm). Finally, the results were expressed as AFU/mg of mycelia dry weight.

## Results

### AtmA influences mitochondrial function plus glucose uptake and/or consumption

**Figure 1:**
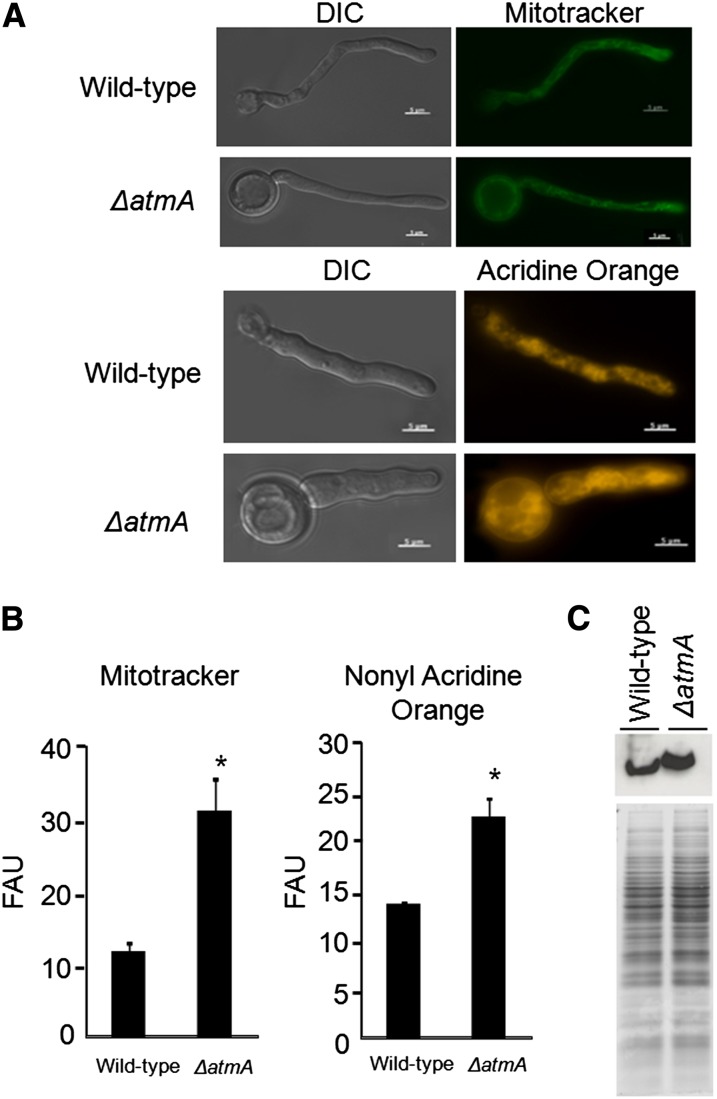
The *ΔatmA* strain has increased mitochondrial mass. (A) Fluorescent microscopy for both wild-type and *ΔatmA* mitochondria stained with Mito Tracker Green and Nonyl Acridine Orange. (B) Flow cytometric analyses (FCA) for both dyes are shown. The results are expressed as mean ± SD and were considered statistically different (*), with *P* < 0.05 determined by Student *t* test using GraphPad Prism software version 5 (GraphPad Software). FAU, fluorescent arbitrary units. (C) Western blot (upper panel) of the cytochrome *c* of the total proteins (lower panel) extracted from the wild-type and *ΔatmA* strains grown for 16 hr in MM.

The *ΔatmA* strain demonstrated elevated ROS accumulation in both nonstarved and starved cells (Figure 7), which could have reflected mitochondrial or respiratory dysfunction. Initially, the speed of oxygen consumption was determined. The absence of *atmA* was shown to impact aerobic respiration efficiencies and resulted in a 50% reduction in oxygen consumption when compared to the wild-type strain (Table 2). Mitochondrial mass in the wild-type and Δ*atmA* strains was subsequently evaluated. Fluorescent microscopy and flow cytometric analyses (FCA) of the mitochondrial stains, Mitotracker Green and NAO, revealed the fluorescence emitted by the Δ*atmA* strain was 3.0-fold and 2.0-fold higher than the wild-type strain using the two respective stains (Figure 1, A and B). One possible explanation for the different mitochondrial staining patterns is that the overall mitochondrial content might be higher in the *ΔatmA* mutant than in the wild-type strain. However, *ΔatmA* germlings have a larger volume, which could contribute to some degree for the higher fluorescent signal. To discard this possibility and test this hypothesis, we quantified the levels of cytochrome *c* in both the wild-type and *ΔatmA* mutant strains by Western blot via densitometry by using the ImageJ software (http://rsbweb.nih.gov/ij/download.html) (Figure 1C). The *ΔatmA* mutant showed approximately two-fold more cytochrome *c* than the wild-type strain (Figure 1C). The reduction in oxygen consumption in the Δ*atmA* strain was therefore not attributed to a reduction in mitochondrial mass. Subsequently, to identify the phase of the oxidative phosphorylation process that was affected by the loss of AtmA function, phase-specific inhibitors were sequentially added and oxygen consumption determined. To evaluate complex IV activity (cytochrome *c* oxidase), complex III and the alternative oxidase were inhibited by the addition of Antimycin A and SHAM, respectively (Table 2). Inhibition of complex III of the respiratory chain, via the addition of antimycin A, resulted in a 31.5% and 34.5% reduction in the wild-type and Δ*atmA* strains, respectively. The remaining capacity to consume oxygen was lost via the addition of the alternative oxidase inhibitor SHAM (these results were similar for both strains) (Table 2). Subsequently, the addition of complex IV substrates, TMPD and ascorbate, were supplied exogenously, increasing oxygen consumption 8.8-fold and 5.6-fold (Table 2) in the wild-type and Δ*atmA*, respectively. The reduced recovery in oxygen consumption in the Δ*atmA* strain reflects a 40% reduction in complex IV cytochrome *c* oxidase activity.

**Figure 2:**
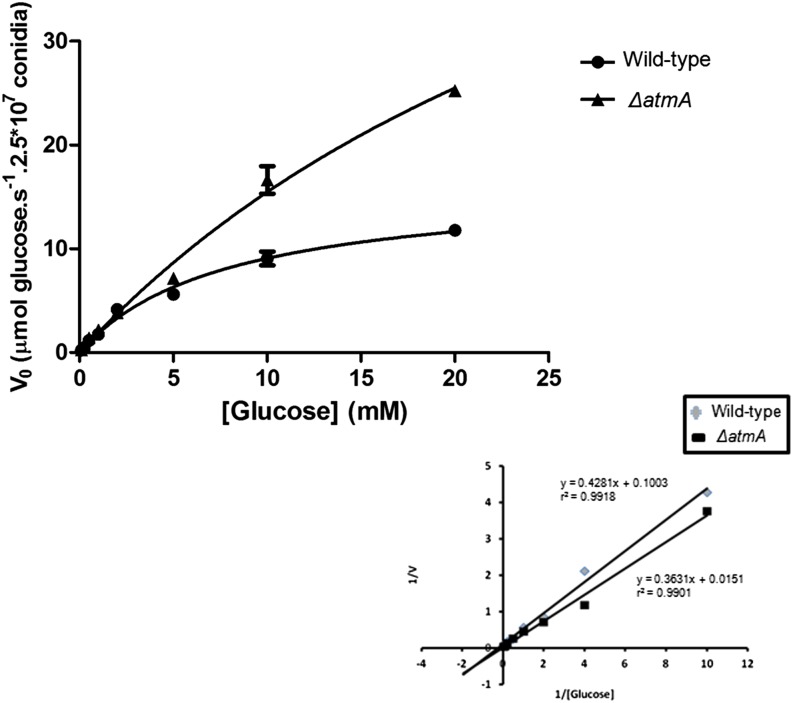
Glucose uptake is impaired in the *ΔatmA* mutant strain. K*_m_* values for glucose in the *A. nidulans* wild-type and *ΔatmA* mutant strains. Uptake rates for [^14^C] glucose germinating conidia of the wild-type and *ΔatmA* mutant strains were determined at the indicated substrate concentrations at pH 7.0. (n = 3; ±SD).

Mitochondrial dysfunction could result in a reduced ability to uptake and utilize glucose. The absence of *atmA* was subsequently revealed to impact glucose uptake. Typical Michaelis-Menten saturation kinetics for glucose uptake was observed for both the wild-type and *ΔatmA* strains (Figure 2). The wild-type strain showed a Km of 7.83 µM and V_max_ of 14.96 ± 1.25 µM glucose s^−1^, whereas the *ΔatmA* strain demonstrated Km values of 36.76 µM, and V_max_ value of 60.87 ± 11.48 µM µM glucose s^−1^, per 2.5×10^7^ conidia. The higher Km and V_max_ for the *ΔatmA* strain indicated a reduced ability to uptake glucose. Collectively, these results strongly indicate that AtmA is important for mitochondrial function and influences aerobic respiration via the cytochrome *c* oxidase and glucose uptake and/consumption.

### Transcriptional profiling of carbon-starved and nonstarved cultures elucidated a role for AtmA during starvation

**Figure 3:**
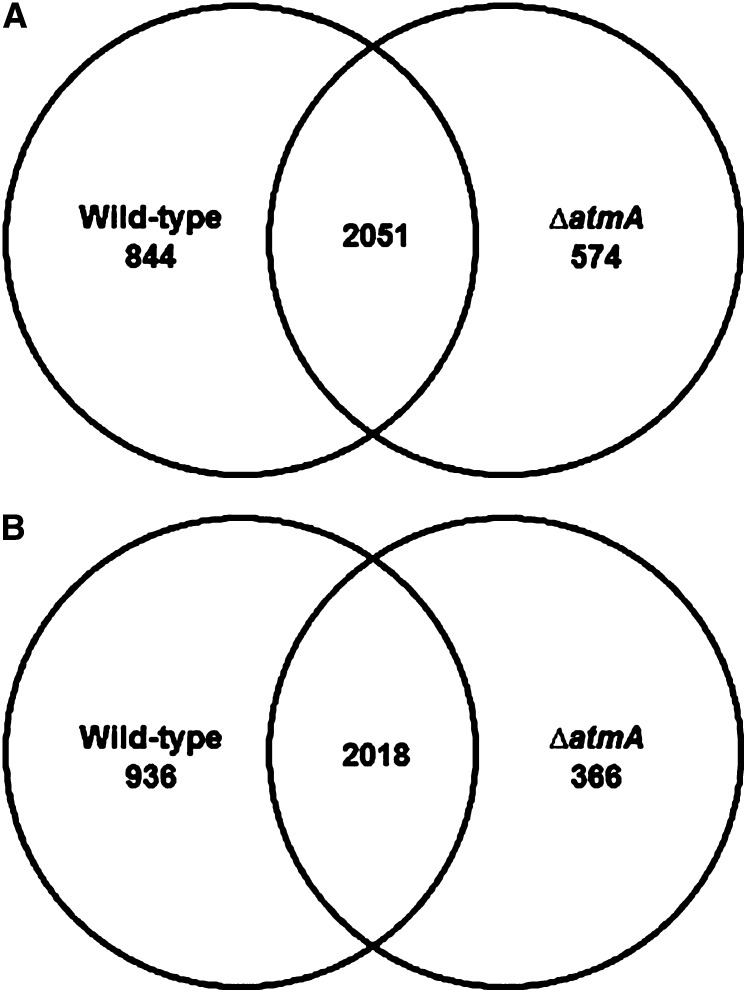
Venn diagrams of the wild-type and Δ*atmA* transcriptomes. The overlap of genes exhibiting a statistically significant (*P* < 0.001) increase (A) or decrease (B) in expression after carbon starvation compared to the equivalent transcriptomes when grown on glucose containing media.

**Figure 4:**
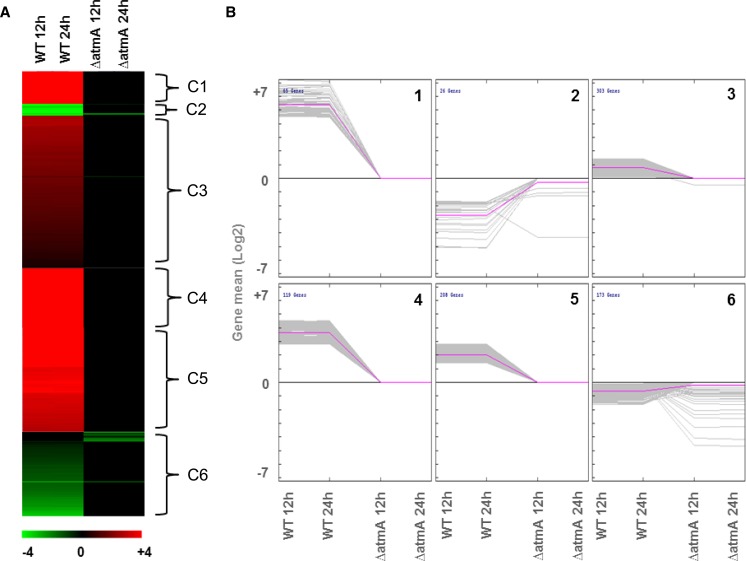
Expression pattern of the genes differentially expressed between the wild-type and Δ*atmA* strains after carbon starvation. (A) Heat map of the six gene clusters (C1 to C6) identified via KMC analysis as demonstrating a similar expression pattern. (B) Expression profile (mean log_2_ fold change) of the genes within the six clusters.

Genome-wide transcriptional profiling of the *ΔatmA* and wild-type strains in the presence and absence of a carbon source was undertaken to investigate the role of AtmA during aerobic respiration and the carbon starvation response. The two strains were grown for 24 hr in MM plus 2% glucose and then transferred to MM without any carbon source for 12 hr and 24 hr. The data set was deposited at http://www.ncbi.nlm.nih.gov/geo/query/acc.cgi?acc=GSE42732. A very high genome-wide correlation in gene expression was observed between the two time points (R > 0.95) within the individual strains. Subsequently, the time points were combined for further analyses. In the wild-type strain, 2895 and 2954 genes were upregulated or downregulated, whereas 2625 and 2384 genes were upregulated or downregulated in the *ΔatmA* strain, after starvation (Figure S1). The genes specifically modulated in an individual strain were identified (Table S2). The majority of the carbon starvation transcriptional responses were conserved between the two strains (Figure 3). The majority of significant differences between the two strains were represented by an absence of a transcriptional response to carbon starvation in the *ΔatmA* strain, predominantly gene induction (Figure 4 and Table S2).

All the biological processes, cellular components, and molecular functions (GO terms) overrepresented in the transcriptional response to carbon starvation in the wild-type and *ΔatmA* strains were identified (Table S3 and Table S4). In both strains, carbon starvation resulted in a downregulation of mitochondrial functions and aerobic respiration, whereas an upregulation of autophagy, vesicle transport, and protein targeting to the vacuole. However, strain-specific responses represented the focus of this study. Wild-type strain–specific responses included an upregulation of TOR signaling, the actin cytoskeleton, and asexual reproduction (Table 3A and Figure 4). Apart from TOR, the other signaling components identified included the *S. cerevisiae Sit4*, *Tap42*, and *Lst8* homologs (AN0120, AN0504, AN1335) and the heat-shock transcription factor *Hsf1* homolog (AN8035). Conversely, there was a wild-type–specific downregulation of the microtubule cytoskeleton, ion transport, ribosomal biogenesis, and RNA processing (Table 3B and Figure 4).

**Table 3:** The GO terms specifically overrepresented in the list of genes significantly upregulated or downregulated in the wild-type strain after carbon starvation

	GO Term	Description	No. Genes	*P*
A	0019954	Asexual reproduction	37	0.0016
	0031929	TOR signaling cascade	7	0.0015
	0030036	Actin cytoskeleton organization	24	0.0033
	0043687	Posttranslational protein modification	71	0.0006
	0043938	Positive regulation of sporulation	12	0.0034
	0045461	Sterigmatocystin biosynthetic process	15	1.6e-06
	0030427	Site of polarized growth	48	0.0016
	0000145	Exocyst	5	0.0051
	0016787	Hydrolase activity	183	8.3e-05
	0044264	Polysaccharide metabolic process	29	0.0031
B	0006879	Cellular iron ion homeostasis	15	0.0062
	0000226	Microtubule cytoskeleton organization	25	0.0066
	0007017	Microtubule-based process	31	0.0045
	0033753	Establishment of ribosome localization	17	0.0034
	0000054	Ribosomal subunit export from nucleus	17	0.0034
	0042254	Ribosome biogenesis	124	7.1e-14
	0006396	RNA processing	138	5e-08
	0032543	Mitochondrial translation	18	7e-06
	0006412	Translation	70	1.3e-05
	0015101	Cation membrane transporter activity	7	0.0010

For full gene lists, refer to Table S3 and Table S4. A, upregulated; B, downregulated.

The *ΔatmA* strain–specific responses to carbon starvation included an upregulation of genes involved in secretion, the CVT pathway, and ER-to-golgi transport [Table 4A, Figure 4 (clusters C2 and C6), Table S2, and Table S3]. However, existence of a CVT pathway in filamentous fungi is still a controversial topic (Kershaw and Talbot 2009; Talbot and Kershaw 2009; Yanagisawa *et al.* 2013). In addition, there was an upregulation of pre-autophagosomal structures, G1/S transition, the amino acid starvation response, and fatty acid oxidation [Table 4A;, Figure 4 (clusters 2 and C6), Table S2, and Table S3). The *ΔatmA*-specific downregulation of glucose catabolic processes was also observed in addition to the downregulation of multiple mitochondrial components, such as the mitochondrial proton transporting ATP synthase, NADH dehydrogenase/oxidoreductase, respiratory chain complex subunits, heme biosynthetic processes, and the pyruvate dehydrogenase complex [Table 4B (cluster C1, C3 to C5), Table S2, and Table S3]. The described set of functions comprehensively demonstrated the downregulation of glucose utilization and mitochondrial oxidative phosphorylation in the absence of AtmA in concordance with the reduced ability of the *ΔatmA* strain to uptake glucose and consume oxygen.

**Table 4:** The GO terms specifically overrepresented in the list of genes significantly upregulated or down regulated in the *ΔatmA* strain after carbon starvation

	GO Term	Description	No. Genes	*P*
A	0046903	Secretion	13	0.0010
	0034198	Cellular response to amino acid starvation	6	0.0012
	0032258	CVT pathway	10	0.0001
	0000082	G1/S transition of mitotic cell cycle	11	0.0019
	0019395	Fatty acid oxidation	7	0.0003
	0006887	Exocytosis	12	0.0014
	0009063	Cellular amino acid catabolic process	21	0.0007
	0030134	ER to Golgi transport vesicle	15	0.0003
	0005773	Vacuole	43	0.0007
	0031982	Vesicle	29	0.0010
	0042175	Nuclear outer membrane–endoplasmic reticulum membrane network	31	0.0015
	0005789	Endoplasmic reticulum membrane	29	0.0027
	0000407	Pre-autophagosomal structure	7	0.0031
	0000139	Golgi membrane	15	0.0068
B	0006122	Mitochondrial electron transport, ubiquinol to cytochrome c	6	0.0042
	0006783	Heme biosynthetic process	9	0.0019
	0015986	ATP synthesis coupled proton transport	10	0.0019
	0006007	Glucose catabolic process	17	0.0026
	0019674	NAD metabolic process	9	0.0046
	0033615	Mitochondrial proton-transporting ATP synthase complex assembly	5	0.0027
	0045039	Protein import into mitochondrial inner membrane	6	0.0042
	0042719	Mitochondrial intermembrane space protein transporter complex	4	0.0087
	0005750	Mitochondrial respiratory chain complex III	6	0.0042
	0045275	Respiratory chain complex III	6	0.0042
	0000275	Mitochondrial proton-transporting ATP synthase complex, catalytic core F(1)	4	0.0087
	0005751	Mitochondrial respiratory chain complex IV	6	0.0042
	0045254	Pyruvate dehydrogenase complex	4	0.0086
	0045277	Respiratory chain complex IV	6	0.0042
	0045259	Proton-transporting ATP synthase complex	10	0.0019
	0016651	Oxidoreductase activity, acting on NADH or NADPH	18	0.0001
	0050136	NADH dehydrogenase (quinone) activity	9	0.0007
	0016655	Oxidoreductase activity, acting on NADH or NADPH, quinone as acceptor	10	0.0002
	0008137	NADH dehydrogenase (ubiquinone) activity	9	0.0007
	0016491	Oxidoreductase activity	140	1.9e-07
	0046961	Proton-transporting ATPase activity, rotational mechanism	10	0.0002
	0003954	NADH dehydrogenase activity	9	0.0007
	0008121	Ubiquinol–cytochrome-c reductase activity	7	0.0014
	0016681	Oxidoreductase activity, acting on diphenol, cytochrome as acceptor	7	0.0014

For full gene lists, refer to Table S3 and Table S4. A, upregulated; B, downregulated.

The reduced ability of the *ΔatmA* strain to utilize glucose was accompanied by an absence of wild-type upregulation in alternative carbon utilization, such as polysaccharides and fatty acids (Table 3, A and B, and Figure 4). The absence of starvation-induced hydrolases transcription, particularly xylosidases, in the *ΔatmA* strain demonstrated the role played by AtmA in the induction of hydrolase secretion, possibly scavenging for any available carbon source (Figure S2).

Collectively, the transcriptomic analysis demonstrated the differential sensing of nutrition/cellular energetic status, plus a reduction in glucose utilization and respiration. A role for AtmA in the carbon starvation–induced transcription of hydrolases also was identified.

### Autophagy is decreased in *ΔatmA* mutant strain

Autophagy forms an integral part of the carbon starvation response in fungi. Transcriptomic analyses showed that autophagy was significantly induced in both the wild-type and *ΔatmA* strains after carbon starvation. However, multiple autophagic processes, such as TOR signaling, the CVT pathway, and pre-autophagosomal structures, were differentially expressed in a strain-specific manner. Subsequently, the influence of AtmA on carbon starvation–induced autophagy in *A. nidulans* was evaluated. The cellular localization of *AtgH^ATG8^*::*GFP*, which is recruited to autophagosomes and transported to the vacuole in response to starvation, was monitored in the wild-type and *ΔatmA* strains. A *ΔatgA^ATG1^ AtgH^ATG8^*::*GFP* double mutant, which also lacked the protein kinase required for vesicle formation during autophagy and the CVT pathway, was utilized as a negative control.

**Figure 5:**
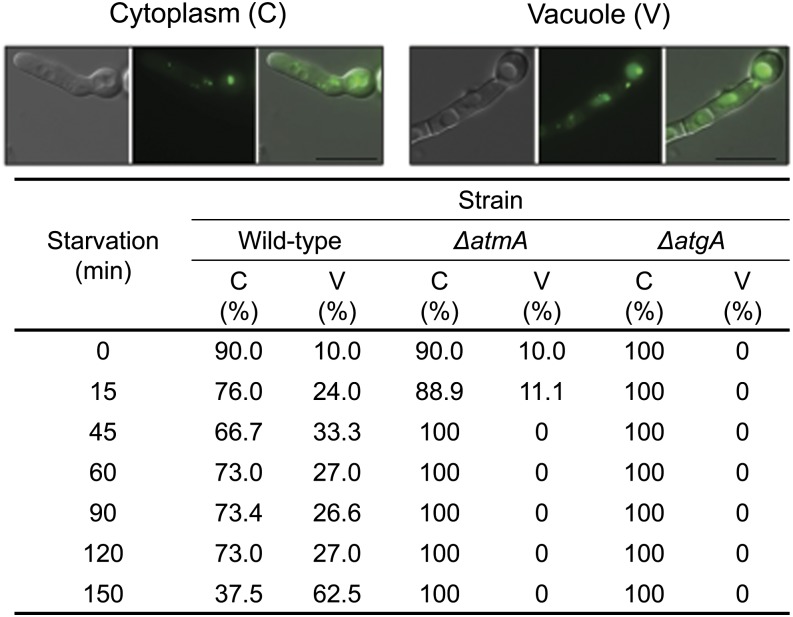
The *atmA* positively controls autophagy. *A. nidulans AtgH::GFP* germlings previously grown for 12 hr in MM plus 2% glucose were transferred to MM without any carbon source for 15, 30, and 60 min. Bars: 10 µm. The *AtgH*::*GFP*, *ΔatmA AtgH*::*GFP*, and *ΔatgA AtgH*::*GFP* grown for 12 hr in MM plus 2% glucose (time zero) and transferred to MM without any carbon source for 15, 45, 60, 90, 120, and 150 min. Bars: 10 µm.

Initially, to confirm that the *atg1* homolog in *A. nidulans*, AtgA, was essential for autophagy, the translocation of AtgH::GFP to the vacuole upon carbon starvation in the *ΔatgA^ATG1^ atgH^ATG8^*::*GFP* double mutant was assessed. The *atgH^ATG8^*::*GFP* strain was used as a positive control. Germlings were grown for 12 hr in 2% glucose and then transferred to MM without any carbon source for 15 to 150 min (Figure 5). After 12 hr of growth in the presence of glucose, AtgH::GFP localized to pre-autophagosomal-like structures and in the cytoplasm (Figure 5). After 150 min of starvation, approximately 60% of germlings contained punctuate fluorescent spots that localize to the vacuole (Figure 5). In the *ΔatgA^ATG1^ atgH^ATG8^*::*GFP* strain, the AtgH::GFP signal did not localize to the vacuoles after 150 min (Figure 5). These results indicate that in *A. nidulans* AtgH translocation to the vacuole on carbon starvation is AtgA-dependent.

Subsequently, the translocation of AtgH::GFP to the vacuole upon carbon starvation in the *ΔatmA* background was evaluated. AtgH::GFP translocation to the vacuoles during starvation was dramatically reduced in the absence of AtmA (Figure 5). Interestingly, before starvation, a low level of AtgH::GFP localized in the vacuole similar to that of the wild-type strain. However, during the prolonged periods of starvation vacuolar localization was lost, reminiscent of the *ΔatgA^ATG1^* strain, suggesting that under nonstarving conditions AtmA does not control AtgH localization. Therefore, under carbon starvation, autophagy appears to be regulated by AtgA^*ATG1*^ and AtmA.

### *A. nidulans* AtmA interacts genetically with XprG on carbon starvation

**Figure 6:**
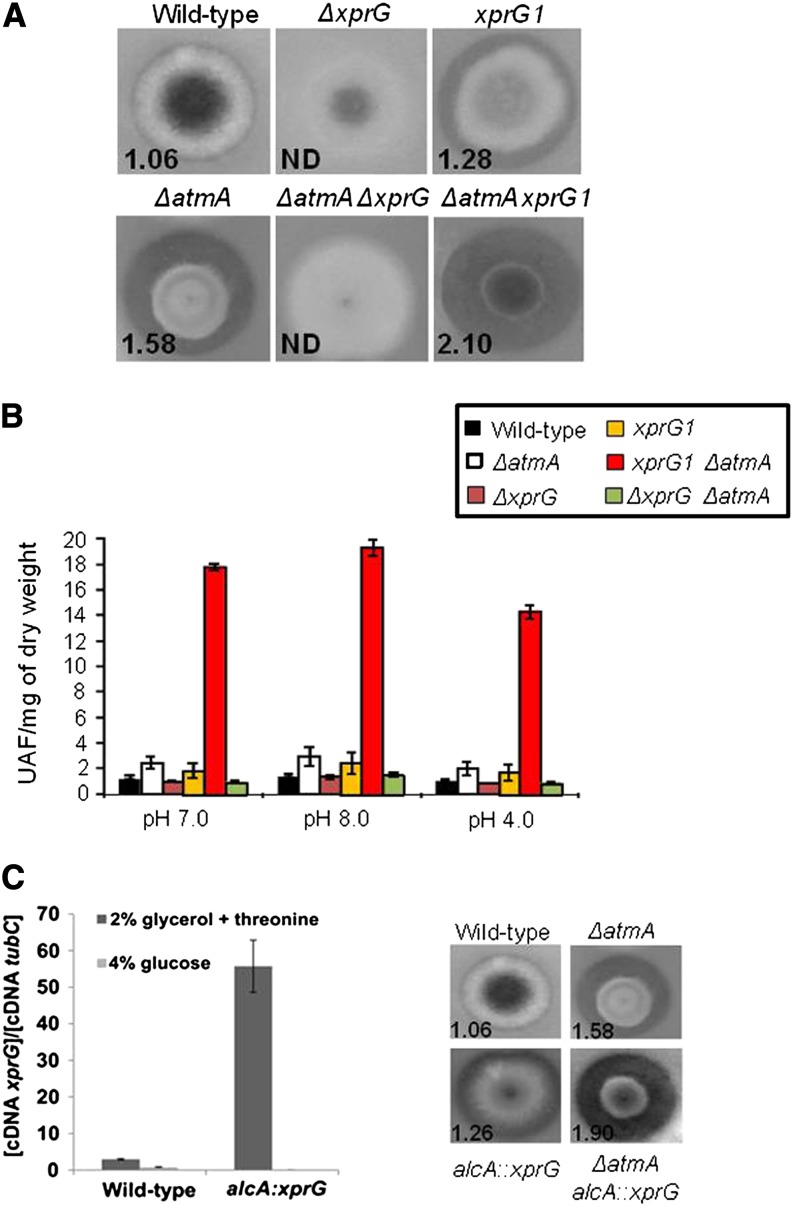
AtmA genetically interacts with *XprG*. (A) The semi-quantitative determination of protease secretion after growth on MM plus milk as the sole carbon source for the wild-type, *ΔatmA*, *ΔxprG*, *xprG1*, *ΔatmA ΔxprG*, and *ΔatmA xprG1* mutant strains. (B) Quantitative evaluation of protease activity of 48-hr carbon-starved cultures for the wild-type, *ΔatmA*, *ΔxprG*, *xprG1*, *ΔatmA ΔxprG*, and *ΔatmA xprG1* mutant strains. (C) Left panel shows real-time RT-PCR for the *xprG* gene. The wild-type and *alcA*::*xprG* mutant strains were grown in liquid MM containing either 2% glycerol and 100 mM threonine or 4% glucose for 24 hr at 37°C. Right panel shows the semi-quantitative determination of protease secretion after growth on MM plus milk as the sole carbon source supplemented with 10 mM cyclopentanone for the wild-type, *alcA*::*atmA*, and *ΔatmA alcA*::*xprG* mutant strains. The numbers on the left bottom side of each panel in (A) and in (B) represent the clearance index (clearance zone diameter/ colony diameter).

*A. nidulans* secretes proteases in an XprG-dependent manner during carbon starvation irrespective of the presence of proteins (Katz *et al.* 1996). Protease secretion was assessed via determining the size of a clearance zone surrounding the fungal colony on agar plates that contained milk as a sole carbon source (Figure 6A) and the clearance index (CI; clearance zone diameter / colony diameter) was calculated. The single *ΔxprG* and double *ΔatmA ΔxprG* mutant strains were confirmed to have a diminished clearance zone (CIs undetermined). The *ΔatmA* strain, however, demonstrated increased secretion or protease activity (CI, 1.58) compared to the wild-type strain (CI, 1.06), despite the fact that as a class of proteins overall proteolytic enzyme transcription was not comparatively increased in the *ΔatmA* strain. However, difference in individual protease transcription could have been sufficiently contributed to the observed phenotype. Similarly, the xprG1 gain-of-function mutation demonstrated increased protease activity(CI,1.28). Interestingly, the double *ΔatmA xprG1* mutant produced a larger clearance zone (CI, 2.10) than that of either the *ΔatmA* or *xprG1* single mutants.

To clarify this genetic relationship, we utilized a more quantitative method to assess protease activity. Because the specificity of the extracellular proteases was not known, we used a synthetic FRET peptide library Abz (or MCA)-GXXXXXQ-EDDnp and Abz (or MCA)-GXXZXXQ-EDDnp. For all strains, no significant differences were seen in the extracellular protease activity in three different pH conditions tested (pH 4.0, 7.0, and 8.5) (Figure 6B). Both the *ΔxprG* and *ΔatmA ΔxprG* mutant strains demonstrated comparable protease activity to the wild-type strain, whereas both the *ΔatmA* and *xprG1* demonstrated approximately twice the protease activity (Figure 6B). The protease activity was six-fold to seven-fold higher in the *ΔatmA xprG1* double mutant (Figure 6B). Therefore, the FRET assay corroborated the previous result observed using the CI.

We also constructed an *alcA*::*xprG* conditional mutant for *xprG* by replacing the *xprG* promoter with the *alcA* promoter. The *alcA* promoter is repressed by glucose, derepressed by glycerol, and induced to high levels by ethanol, L-threonine, or cyclopentanone (Flipphi *et al.* 2002). Transformants were selected that accumulated approximately 13-fold higher *xprG* mRNA when transferred to glycerol 2% plus threonine than when transferred to glucose 4% (Figure 6C, left panel). An increased clearance zone around the *alcA*::*xprG* mutant colony was observed when grown on agar plates containing milk supplemented with 10 mM cyclopentanone (CI, 1.26) (Figure 6C, right panel). Again, to examine the possible genetic interactions between *atmA* and *xprG*, a double *ΔatmA alcA*::*xprG* mutant was constructed. The clearance zone of the *ΔatmA alcA*::*xprG* strain (CI, 1.90) (Figure 6C, right panel) was larger than that produced by the single *alcA*::*xprG* mutant. These results imply that AtmA performs a role in the inhibition of XprG.

**Figure 7:**
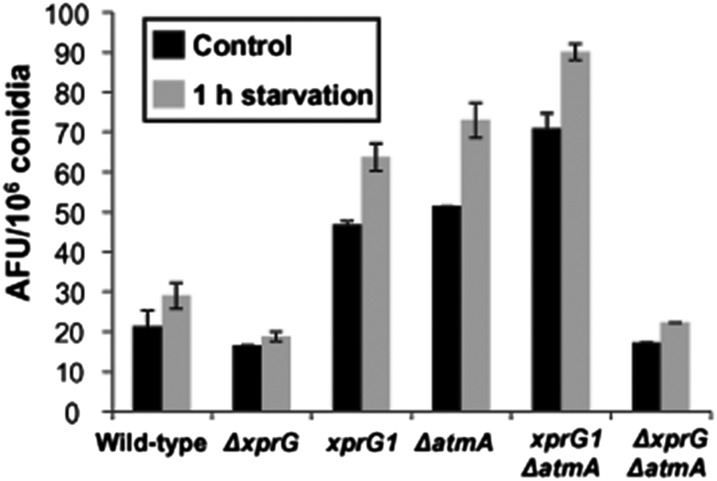
ROS production by *ΔatmA* and *ΔxprG* mutant strains. Intracellular ROS levels after 1-hr carbon starvation determined via the oxidant-sensitive probe 5-(and 6)-chloromethyl-2′,7′-dichlorofluorescin diacetate CM-H_2_DCFDA. AFU, arbitrary fluorescence units.

The accumulation of ROS in the *ΔatmA* strain, when grown in the presence of glucose or after carbon starvation, was twice that observed for the wild-type strain (Figure 7). Accumulation of ROS in strains lacking *xprG* was moderately less than that of the wild-type strain, whereas the gain-of-function *xprG1* strain demonstrated ROS levels comparable to the *ΔatmA* strain. Similar to the protease results previously presented, the combination of the *ΔatmA* and *xprG1* mutations in a single strain demonstrated an additive effect and increased ROS accumulation to the highest levels. These results suggest that under these conditions, XprG is epistatic and is needed for the increased production of ROS caused by the loss of AtmA.

**Figure 8:**
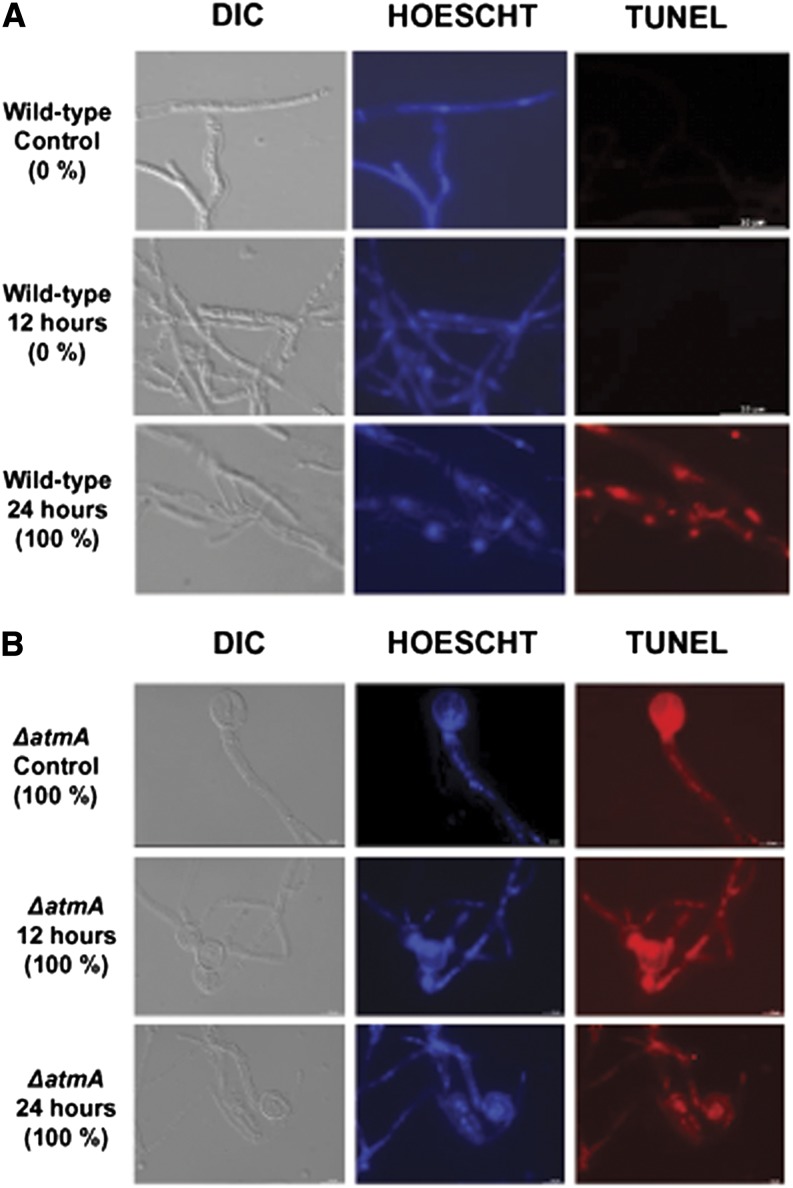
TUNEL assay to detect DNA fragmentation in the wild-type and *ΔatmA* strains evaluating the influence of 0, 12, and 24 hr of starvation. Nuclei were visualized by Hoescht staining. (A) Wild-type and (B) *ΔatmA* mutant strains. Bars: 10 µm.

**Figure 9:**
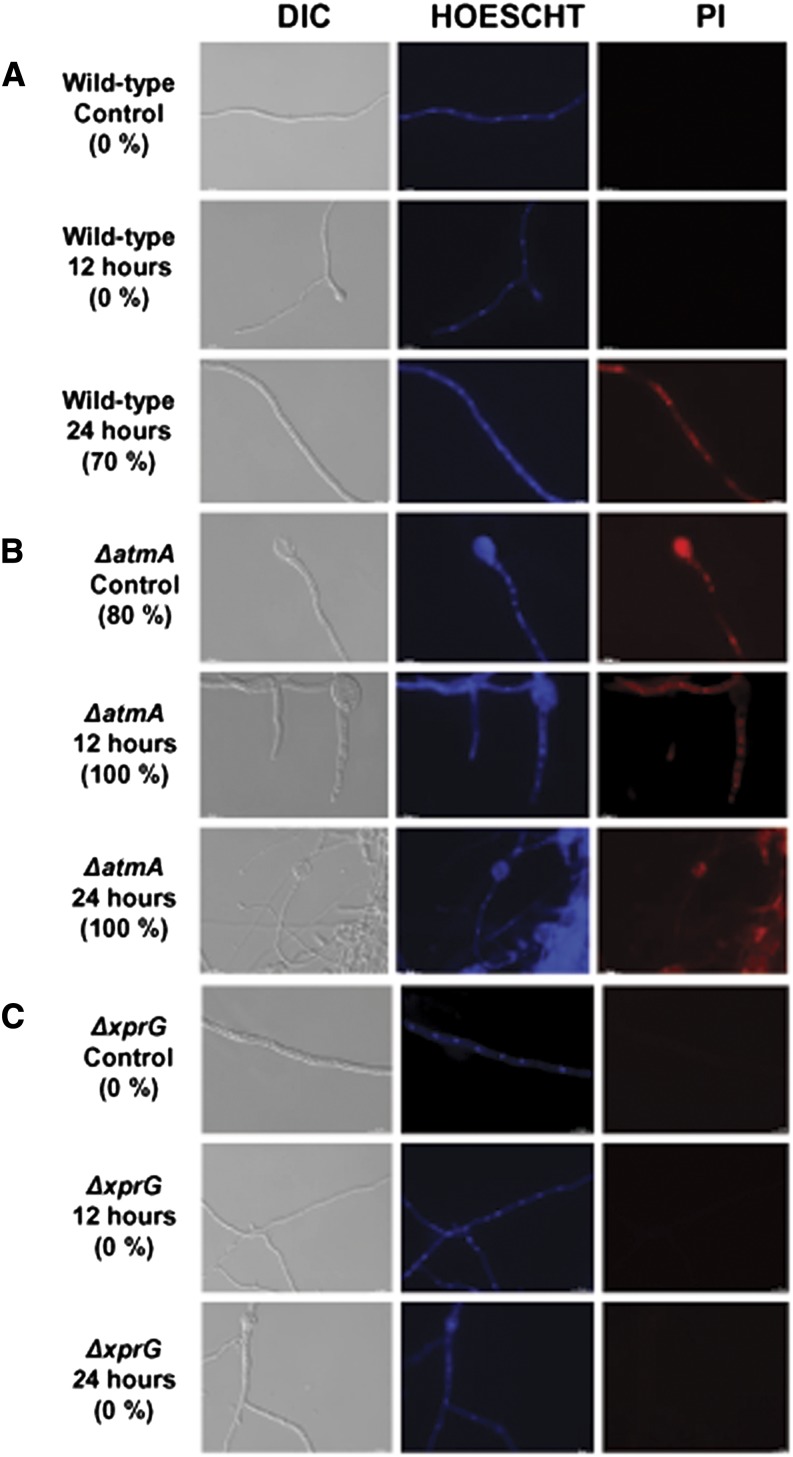
Detection of necrosis using propidium iodide (PI) staining for wild-type, *ΔatmA*, and *ΔxprG* strains after 0, 12, and 24 hr of starvation. Nuclei were visualized by Hoescht staining. (A) Wild-type, (B) *ΔatmA*, and (C) *ΔxprG*. Bars: 10 µm.

Although the *ΔatmA* mutant is viable, it shows nuclei with considerable DNA fragmentation (Malavazi *et al.* 2006, 2008). The AtmA^ATM^ loss-of-function caused synthetic lethality when combined with mutation in UvsB^ATR^, suggesting that AtmA and UvsB interact and are probably partially redundant in terms of DNA damage sensing or repairing and polar growth (Malavazi *et al.* 2008). This functional redundancy could help to explain why *ΔatmA* mutant is viable despite all the DNA fragmentation. Thus, a combination of TUNEL and propidium iodide (PI) staining, which respectively identified DNA fragmentation or a loss of cell membrane integrity, was utilized to monitor apoptosis and necrosis upon carbon starvation (Figure 8 and Figure 9, respectively). Before 24-hr carbon starvation, the wild-type strain showed no signs of apoptosis or necrosis. However, after 24-hr starvation, 100% and 70% of wild-type hyphae demonstrated positive TUNEL and PI staining, respectively. In contrast, all nonstarved or starved *ΔatmA* hyphae stained positively with TUNEL, whereas 80% and 100% of hyphae stained positively with PI under nonstarved and starved conditions, respectively. The *ΔxprG* strain showed no TUNEL or PI staining in nonstarved or starved hyphae. The *ΔatmA ΔxprG* double mutant demonstrated no staining for the nonstarved culture and approximately 50% TUNEL and PI nuclear staining after starvation. Collectively, these data demonstrate that under starvation, AtmA inhibits XprG-dependent and XprG-independent forms of inducing cell death.

## Discussion

Mitochondria supply cellular energy but also perform a role in the adaptation to stress and the cross-talk between prosurvival and prodeath pathways. ATM phosphorylates more than 700 proteins, including many downstream kinases (Matsuoka *et al.* 2007) demonstrating a broad range of functions. ATM is known for its role in the DNA damage response and cell cycle in mammalian and fungal cells (Ditch and Paull 2012; Malavazi *et al.* 2006, 2007). ATM also has recently been shown to be the redox sensor that controls mitochondrial and metabolic function through the action of the downstream kinases AMPK and mTOR (Ditch and Paull 2012). Additionally, *Atm*-deficient mice cells revealed increased mitochondrial mass, altered morphology, and deficiencies in mitochondrial electron transport chain activity correlating to impaired ATP levels and elevated mitochondrial ROS (Valentin-Vega and Kastan 2012). In the presented study of *A. nidulans*, the fungal ATM homolog was shown to control mitochondrial mass and function, which directly or indirectly influenced glucose uptake while also influencing cell death in response to carbon starvation. Moreover, the increased mitochondrial mass in *ΔatmA* cells may not result from changes in mitochondrial biogenesis, but rather from defects in the selective clearance of damaged mitochondria because autophagy is also affected in this strain, in agreement with the work of Valentin-Vega and Kastan (2012). Subsequently, transcriptomic and genetic studies were utilized to elucidate the role played by AtmA during starvation.

The retrograde response signals mitochondrial dysfunction to the nucleus, reconfiguring expression, including an induction of gluconeogenesis, the glyoxylate cycle, and glutamate biosynthesis (Jazwinski 2013). The intrinsic induction of cell death, caused by energetic failure, oxidative stress, and lipid metabolism abnormalities, is mainly regulated by the mitochondria and involves the stress-mediated release of cytochrome C (Zdralevic *et al.* 2012). Inhibition of TOR can induce autophagy and the retrograde response (Zdralevic *et al.* 2012). Increased glyoxylate cycle activity occurs in *S. cerevisiae* during severe nutrient limitation (Wang *et al.* 2010). Fatty acid β-oxidation, which is required to generate acetate to fuel the glyoxylate cycle, was shown to be regulated by AtmA during starvation, suggesting that AtmA performs a role in the retrograde response. In addition, during starvation, TOR and the *S. cerevisiae Sit4*, *Tap42*, and *Lst8* homologs (AN0120, AN0504, AN1335) were shown to be directly or indirectly influenced by AtmA. In *S. cerevisiae* Sit4 and Tap42, which associate with the protein phosphatase 2A, are regulated by TOR and control G1/S cell-cycle transition, whereas Lst8 is a known retrograde response regulator (Jazwinski 2013; Zdralevic *et al.* 2012). The homolog of the *Hsf1* heat-shock transcription factor (AN8035), which is hyperphosphorylated by Snf1 (fungal AMPK homolog) in carbon-starved *S. cerevisiae* cells (Hahn and Thiele 2004), was also influenced by AtmA. Therefore, in response to metabolic stress, AtmA may perform a role in the regulation of TOR signaling that involves the retrograde response and the SnfA pathways. This hypothesis is supported by the observation that the *ΔatmA* strain does not successfully undergo autophagy during carbon starvation, a process known to be controlled by TOR. This suggests that a link between AtmA, SnfA, and TOR may also exist in *A. nidulans*, as observed in mammalian cells.

Recently, AtmA has been demonstrated to perform a role in the regulation of hydrolytic enzyme production (Brown *et al.* 2013). However, nuclear localization of the carbon catabolite repressor, CreA, in the *ΔatmA* strain was similar to the parental strains (Brown *et al.* 2013). On carbon starvation, *A. nidulans* induces the transcription of alternative carbon source usage genes, including carbohydrate active enzymes (CAZy), a response that is dependent on the SnfA and SchA (Brown *et al.* 2013). The absence of AtmA resulted in a loss of starvation-induced CAZy enzyme induction. Therefore, AtmA forms part of a starvation response that influences hydrolytic enzyme transcription and secretion while not being involved in CreA derepression. This supports the hypothesis that other pathways for SnfA and SchA induction exist.

Autophagy is a highly conserved process in eukaryotic cells and occurs in response to a diverse array of conditions, including nutrient deprivation (Martinet *et al.* 2006; Mizushima and Klionsky 2007). Carbon-starved *A. nidulans* downregulated mitochondrial functions and aerobic respiration while upregulating autophagy, vesicle transport, and protein targeting to the vacuole. A dramatic reduction in autophagy was observed in the *ΔatmA* strain, strongly suggesting that AtmA positively regulates autophagy. Autophagy forms part of a starvation-induced prosurvival strategy, which is inhibited by the action of TOR, PKA, and Sch9 on the Atg1 kinase in *S. cerevisiae* (Kamada *et al.* 2000; Yorimitsu *et al.* 2007). In *A. nidulans*, AtgA^ATG1^ was confirmed to be essential for the induction of autophagy, whereas the absence of AtmA resulted in the loss of AtgH^ATG8^ vacuole localization and impaired autophagy. Therefore, the reduction in autophagy in the *ΔatmA* strain could have contributed to the faster rate of starvation-induced cell death.

In mammalian cells, p53 is a multi-functional protein that simultaneously regulates distinct downstream pathways controlling antioxidant defense, cell-cycle progression, mitochondrial respiration, glucose homeostasis, and apoptosis (Ko and Prives 1996; Levine 1997; Armata *et al.* 2010). Different residues of the p53 protein are phosphorylated by ATM on exposure to distinct stimuli (Bensimon *et al.* 2011; Ditch and Paull 2012). In response to DNA damage and during the regulation of glucose homeostasis, ATM phosphorylates p53, resulting in its activation and an increase in protein stability (Shiloh 2006; Armata *et al.* 2010). In mammalian cells, p53-induced autophagy has been reported (Inoki *et al.* 2003; Zhao and Klionsky 2011). In *A. nidulans*, autolysis and starvation-induced protease secretion are controlled by the p53-like transcription factor XprG (Katz *et al.* 2006, 2009). The present study suggests that AtmA regulated the XprG-dependent starvation-induced protease secretion and cell death.

ROS act as signaling molecules and cellular toxicants. Mitochondrial dysfunction or the impairment of oxidative phosphorylation results in ROS accumulation (Barros *et al.* 2004). A shift in the balance between oxidants/antioxidants in the direction of oxidation contributes to the induction of apoptosis (Liu *et al.* 2008; Giorgio *et al.* 2007). In mammalian cells, p53 mediates antioxidant pathways and thereby protects against endogenous ROS. After stress, high levels of p53 lead to a shift in oxidant/antioxidant balance, which can result in apoptotic cell death (Kang *et al.* 2013). *Atm*-deficient mammalian cells demonstrate increased ROS levels and oxidative stress (Barlow *et al.* 1999; Gatei *et al.* 2001; Kamsler *et al.* 2001), with both conditions resulting in the phosphorylation of p53 at the ATM site (Armata *et al.* 2010). In *A. nidulans*, AtmA negatively regulated XprG-dependent ROS accumulation. Therefore, the absence of AtmA or XprG would result in faster and slower rates in ROS-mediated cell death in the respective single-gene deletion strains. This suggests that AtmA negatively regulates XprG, as demonstrated by the increase in XprG-dependent secretion of proteases, accumulation of ROS, and cell death. This concept is supported by the observed higher level of protease secretion and ROS accumulation in both the Δ*atmA xprG1* (gain-of-function) and the *ΔatmA alcA::XprG* overexpression double mutant strains. The interaction between ATM and p53 has been extensively studied in mammalian systems, in which the interaction between ATM and p53 regulates the G1/S cell-cycle checkpoint, apoptosis, or senescence through p53 phosphorylation at Ser15 residue (Barlow *et al.* 1997; Banin *et al.* 1998; Bartkova *et al.* 2006; Canman *et al.* 1998; Zhan *et al.* 2010). The mammalian p53 has four ATM-dependent phosphorylation sites (Saito *et al.* 2002; Armata *et al*, 2010; File S1). The *A. nidulans* XprG only has two serine residues at positions 18 and 46 (File S1). The functionality of these residues in XprG and if AtmA phosphorylates them remains to be determined.

Mitochondria are crucial to metabolism, cell-cycle progression, signaling, and apoptosis. One of the striking aspects of the ATM-dependent pathology is that the mitochondria leads to inefficient respiration and energy metabolism plus the increased generation of free radicals that are able to create life-threatening DNA lesions (Ambrose and Gatti 2013). We have observed an apparent discrepancy between the increased mitochondrial copy number in the *A. nidulans ΔatmA* mutant and defects in glucose uptake and oxygen consumption in this mutant. Valentin-Vega *et al.* (2012) have shown that Atm loss also leads to an elevated mitochondrial mass. These authors investigated the dynamics of this organelle and suggested that the increase in mitochondrial mass in Atm-null cells does not result from changes in mitochondrial biogenesis, but rather from defects in the selective clearance of damaged mitochondria by autophagy (mitophagy). We observed decreased autophagy in the *A. nidulans ΔatmA* mutant, therefore it is possible the increased mitochondrial mass in this mutant would reflect a decreased mitophagy as observed in mammalian Atm cells. In response to severe carbon limitation, the retrograde response communicates mitochondrial dysfunction to the cell nucleus, resulting in stress adaptations. The mammalian ATM kinase acts as a redox sensor, coordinating such stress responses via the mTOR, AMPK, and p53 pathways. In *A. nidulans*, AtmA also regulated glucose utilization and mitochondrial oxidative phosphorylation. Carbon starvation responses, including autophagy, shifting metabolism to the glyoxylate cycle and the secretion of carbon scavenging enzymes were AtmA-dependent. Thus, AtmA may represent a link between mitochondrial function and metabolic output with cell cycle and growth, possibly through the influence of the TOR and XprG signaling.

## Supplementary Material

Supporting Information
