# Supplementary material for: The Aspergillus nidulans ATM Kinase Regulates Mitochondrial Function, Glucose Uptake and the Carbon Starvation Response
Source: G3 (Bethesda). 2013 Nov 5;4(1):49–62. doi: 10.1534/g3.113.008607 (PMC3887539; doi:10.1534/g3.113.008607)
Supplement: Supporting Information [file supp_4_1_49__index.html]

The Aspergillus nidulans ATM Kinase Regulates Mitochondrial Function, Glucose Uptake and the Carbon Starvation Response — Supporting Information 

# The *Aspergillus nidulans* ATM Kinase Regulates Mitochondrial Function, Glucose Uptake and the Carbon Starvation Response

## Supporting Information for Krohn *et al.*, 2014

**Files in this Data Supplement:**

- Supporting Information - Figures S1-S2, File S1, and Tables S1-S4 (PDF, 719 KB)
- Figure S1 - Comparison of the genome-wide transcriptional profile post transfer from glucose containing media (represented by a gene mean of zero) to carbon starvation (log2 fold change). (PDF, 454 KB)
- Figure S2 - The absence of a starvation-induced increase in hydrolase transcription in the Δ*atmA* strain. (PDF, 358 KB)
- File S1 - Comparison between the phosphorylation sites of human p53 and *A. nidulans* XprG. (PDF, 517 KB)
- Table S1 - Primers used in this study. (PDF, 344 KB)
- Table S2 - The list of *A. nidulans* genes significantly modulated upon carbon starvation (p<0.001) in either the wild-type, Δ*atmA* or both strains. (.xlsx, 74 KB)
- Table S3 - Distribution of the genes in the subclusters of the hierarquical clustering. (.xlsx, 544 KB)
- Table S4 - The overrepresented GO terms (Fisher's exact test, p<0.05) in the list of genes significantly modulated in the wild-type (WT) and Δ*atmA* strains in response to carbon starvation. (.xls, 82 KB)
