# Supplementary material for: The Aspergillus nidulans ATM Kinase Regulates Mitochondrial Function, Glucose Uptake and the Carbon Starvation Response
Source: G3 (Bethesda). 2013 Nov 5;4(1):49–62. doi: 10.1534/g3.113.008607 (PMC3887539; doi:10.1534/g3.113.008607)
Supplement: Supporting Information [file supp_g3.113.008607_008607SI.pdf]

G3: Genes | Genomes | Genetics

The *Aspergillus nidulans* ATM kinase (AtmA) regulates mitochondrial function, glucose uptake and the carbon starvation response

Nadia Graciele Krohn[†*], Neil Andrew Brown[†*], Ana Cristina Colabardini[†], Thaila Reis[†], Marcela Savoldi[†], Taísa Magnani Dinamarco[††], Maria Helena S. Goldman[††], and Gustavo Henrique Goldman[†,‡]

[‡]Laboratório Nacional de Ciência e Tecnologia do Bioetanol – CTBE, Caixa Postal 6170, 13083-970 Campinas, São Paulo, Brazil and [†]Faculdade de Ciências Farmacêuticas de Ribeirão Preto and [††]Faculdade de Filosofia, Ciências e Letras de Ribeirão Preto, Universidade de São Paulo, São Paulo, Brazil

Corresponding author: Dr. Gustavo H. Goldman
Faculdade de Ciências Farmacêuticas de Ribeirão Preto, Universidade de São Paulo
Av. do Café S/N, CEP 14040-903, Ribeirão Preto, São Paulo, Brazil
Phone/Fax: 55-16-36024280/4281/4311
e-mail address: ggoldman@usp.br

[*]Both authors contributed equally to this manuscript

DOI: 10.1534/g3.113.008607

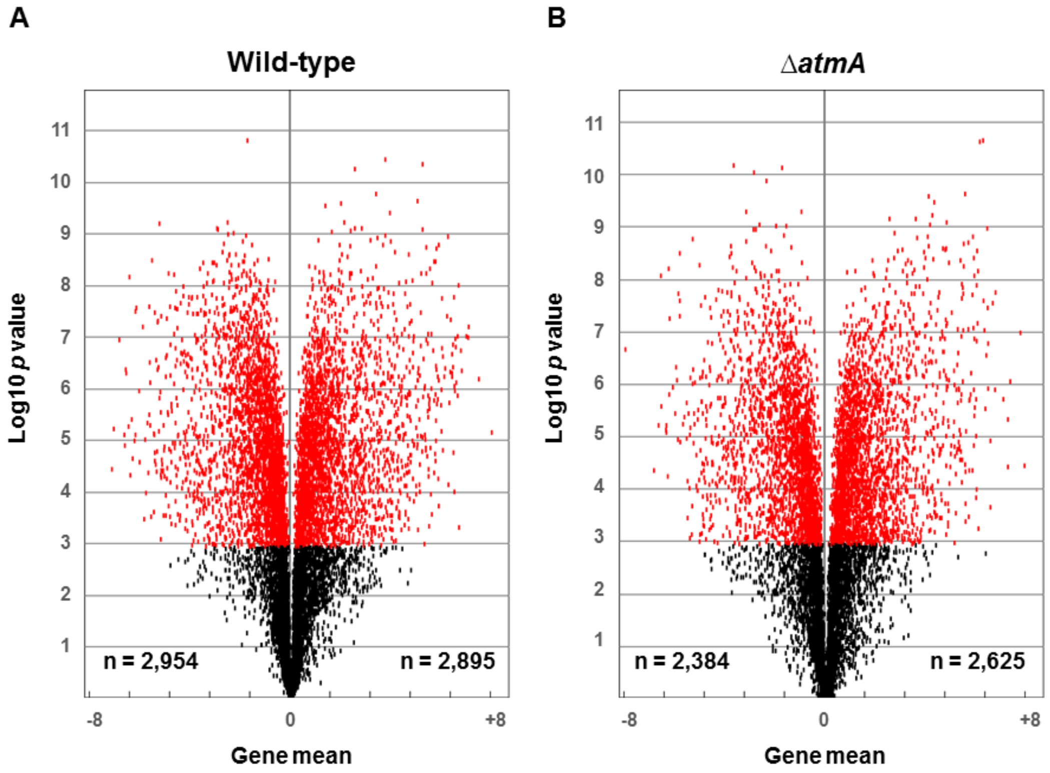

Figure S1 Comparison of the genome-wide transcriptional profile post transfer from glucose containing media (represented by a gene mean of zero) to carbon starvation (log2 fold change). **A)** Wild-type response. **B)** Δ*atmA* response. Red dots indicate genes with significant modulation in expression post starvation. N = number of gene significantly induced or repressed in a particular strain.

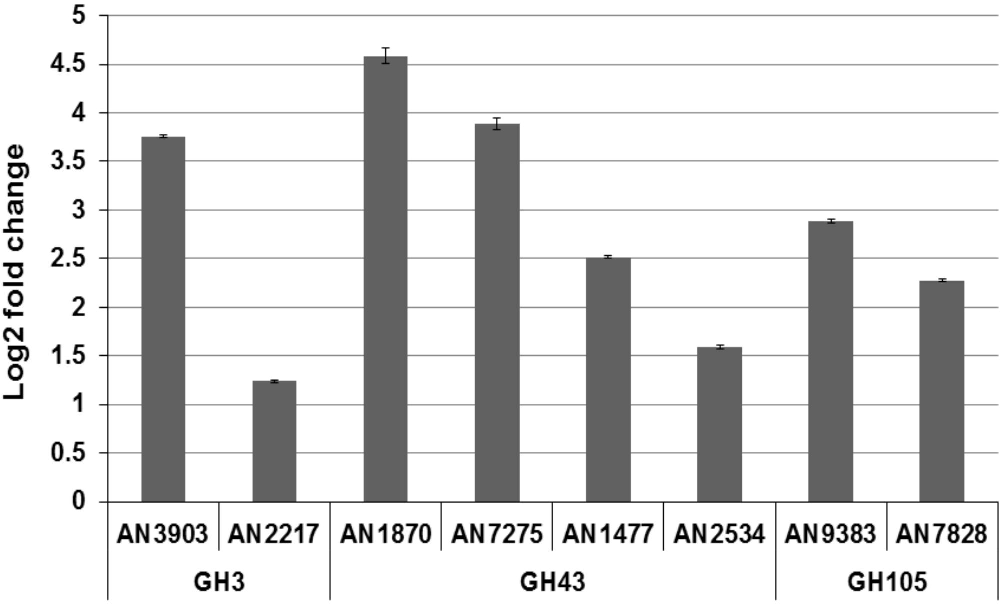

Figure S2 The absence of a starvation-induced increase in hydrolase transcription in the Δ*atmA* strain. Mean log2 fold change in gene expression in the wild-type strain post 12 and 24 h carbon starvation, organised according to CAZy classification. Note that no significant induction of these genes was observed in Δ*atmA* strain.

File S1

Comparison between the phosphorylation sites of human p53 and *A. nidulans* XprG.

Homo sapiens (p53) 393aa [pI 6.37; Mw 43683.16 Da-Expasy Compute pI/Mw tool]

MEEPQSDPSVEPPLSQETFSDLWKLLPENNVLSPLPSQAMDDLMLSPDDIEQWFTEDPGPDEAPRMPEAA
PRVAPAPAAPTPAAPAPAPSWPLSSSVPSQKTYQGSYGFRLGFLHSGTAKSVTCTYSPALNKMFCQLAKT
CPVQLWVDSTPPPGTRVRAMAIYKQSQHMTEVVRRCPHHERCSDSDGLAPPQHLIRVEGNLRVEYLDDRN
TFRHSVVVPYEPPEVGSDCTTIHYNYMCNSSCMGGMNRRPILTIITLEDSSGNLLGRNSFEVHVCACPGR
DRRTEEENLRKKGEPHHELPPGSTKRALSNNTSSSPQPKKKPLDGEYFTLQIRGRERFEMFRELNEALEL
KDAQAGKEPGGSRAHSSHLKSKKGQSTSRHKKLMFKTEGPDSD

Aspergillus nidulans (XprG) 490aa [pI 9.36; Mw 52839.41 Da-Expasy Compute pI/Mw tool]
.................18..........................46
MVTSFDPPPPAEPPADSSIDHNNKLLSFSMPVYPWTLLDYSFRRASISISAQLHGMFFLAESPWTTSPTE
NAPPQQGAELTCYRRNLFQITGSVTLPRALRYIITDTGDRIPIVAHELTVSATESVEGNSVKIISVPWKT
PAANDAGKDTGNSSNTAAKVEKEPPAIPLDMLTGQDLDADYATFPIAWKRLQFRVATANNGRRKELQQHF
VVRLRVVATLSTGMKTPICEVHSGPVIVRGRSPRNFQSRKDLPLSGSAAASRKNAQAAAASNNLTRTSPS
LTDKAKTVVKSSSPETSSNGVPQQSPPNWALATNSTLPPPTTTTLPHSSVYSQSSPEFSRPVEAHRRTTS
....332
AIAAPINLSLLDDDSLNLSNGDSRPHTSFSNDLASKSLSVDSGRPVKMRKVSHSMPQAQSRSTSATFLNT
ANFQQMLPVPFTSESADVLYEYFPLGLEDWQGPVDAVYRPHVVHHTNMPQMKYITARGQSKRYFAAEDVF

Ser9 (in response to ionizing radiation in human p53): absent in *A. nidulans*
Ser15 (G1-S cell cycle checkpoint, apoptosis in human p53): absent in *A. nidulans*
Ser18 (glucose homeostasis in human p53): present in *A. nidulans*
Ser46 (apoptosis in human p53): present in *A. nidulans*

Table S1 Primers used in this study

Primers	Sequence
5’ UTR XprG pRS426 fw	5’-GTAACGCCAGGGTTTTCCCAGTCACGACGGCACTACAATTCCCCAGCCCTT-3’
5’ UTR XprG pyrG rv	5’-GTGCCTCCTCTCAGACAGAATGGCAAGGGTTCGGCTGTA-3’
pyrG alcA fw	5’-GCATTGTTTGAGGCGAATTCTGAAAAGCTGATTGTGATAGTTCC-3’
pyrG alcA fw	5’-GCATTGTTTGAGGCGAATTCTGAAAAGCTGATTGTGATAGTTCC-3’
alcA orf XprG fw	5’-AGTTAATTAGCGGTACCGGGATGGAGGGCTTCGACACC-3’)
pMCB17 alcA rv primers	5’-CCCGGTACCGCTAATTAACT-3’
pRS426 orf XprG rv	5’-GCGGATAACAATTTCACACAGGAAACAGCTCAGACTGGGCGAGGTTCG-3’
TubC Fw	ATGCCGTCGCCGAAAG
TubC Rv	CATTCGGACGAGACATTC
XprG Fw	CGGCTGATCTGGAAAAGATTTCGC
XprG Rv	AAATGCTCCGCCTCAGCAAG

Tables S2-S4 are available for download as Excel files at http://www.g3journal.org/lookup/suppl/doi:10.1534/g3.113.008607/-/DC1.

Table S2 The list of *A. nidulans* genes significantly modulated upon carbon starvation (p<0.001) in either the wild-type, Δ*atmA* or both strains.

Table S3 Distribution of the genes in the subclusters of the hierarquical clustering.

Table S4 The overrepresented GO terms (Fisher's exact test, p<0.05) in the list of genes significantly modulated in the wild-type (WT) and Δ*atmA* strains in response to carbon starvation.
