# Supplementary material for: The Aspergillus nidulans ATM Kinase Regulates Mitochondrial Function, Glucose Uptake and the Carbon Starvation Response
Source: G3 (Bethesda). 2013 Nov 5;4(1):49–62. doi: 10.1534/g3.113.008607 (PMC3887539; doi:10.1534/g3.113.008607)
Supplement: Supporting Information [file supp_g3.113.008607_FigureS1.pdf]

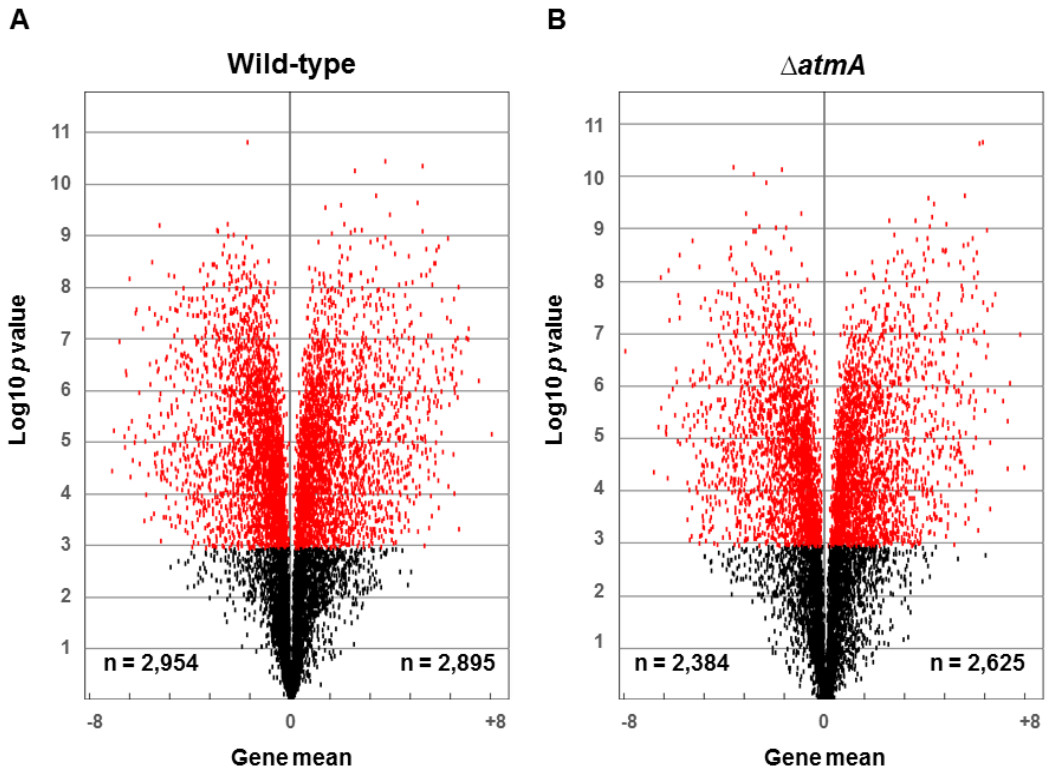

Figure S1 Comparison of the genome-wide transcriptional profile post transfer from glucose containing media (represented by a gene mean of zero) to carbon starvation (log2 fold change). **A)** Wild-type response. **B)** Δ*atmA* response. Red dots indicate genes with significant modulation in expression post starvation. N = number of gene significantly induced or repressed in a particular strain.
