# Supplementary material for: The Aspergillus nidulans ATM Kinase Regulates Mitochondrial Function, Glucose Uptake and the Carbon Starvation Response
Source: G3 (Bethesda). 2013 Nov 5;4(1):49–62. doi: 10.1534/g3.113.008607 (PMC3887539; doi:10.1534/g3.113.008607)
Supplement: Supporting Information [file supp_g3.113.008607_FigureS2.pdf]

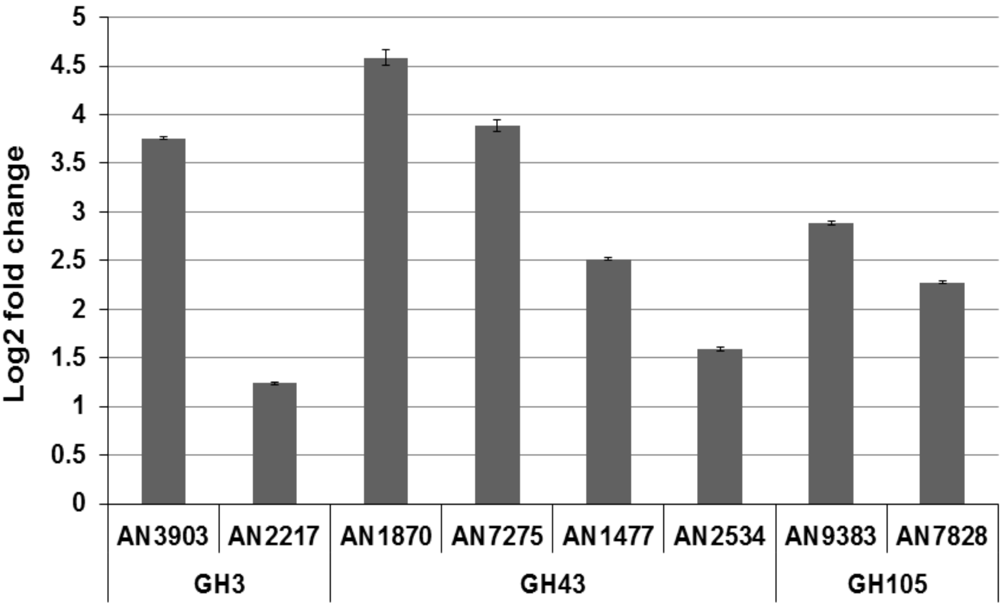

Figure S2 The absence of a starvation-induced increase in hydrolase transcription in the Δ*atmA* strain. Mean log2 fold change in gene expression in the wild-type strain post 12 and 24 h carbon starvation, organised according to CAZy classification. Note that no significant induction of these genes was observed in Δ*atmA* strain.
