# Supplementary material for: The Aspergillus nidulans ATM Kinase Regulates Mitochondrial Function, Glucose Uptake and the Carbon Starvation Response
Source: G3 (Bethesda). 2013 Nov 5;4(1):49–62. doi: 10.1534/g3.113.008607 (PMC3887539; doi:10.1534/g3.113.008607)
Supplement: Supporting Information [file supp_g3.113.008607_FileS1.pdf]

File S1

Comparison between the phosphorylation sites of human p53 and *A. nidulans* XprG.

Homo sapiens (p53) 393aa [pI 6.37; Mw 43683.16 Da-Expasy Compute pI/Mw tool]

```
MEEPQSDPSVEPPLSQETFSDLWKLLPENNVLSPLPSQAMDDLMLSPDDIEQWFTEDPGPDEAPRMPEAA
PRVAPAPAAPTPAAPAPAPSWPLSSSVPSQKTYQGSYGFRLGFLHSGTAKSVTCTYSPALNKMFCQLAKT
CPVQLWVDSTPPPGTRVRAMAIYKQSQHMTEVVRRCPHHERCSDSDGLAPPQHLIRVEGNLRVEYLDDRN
TFRHSVVVPYEPPEVGSDCTTIHYNYMCNSSCMGGMNRRPILTIITLEDSSGNLLGRNSFEVHVCACPGR
DRRTEEENLRKKGEPHHELPPGSTKRALSNNTSSSPQPKKKPLDGEYFTLQIRGRERFEMFRELNEALEL
KDAQAGKEPGGSRAHSSHLKSKKGQSTSRHKKLMFKTEGPDSD
```

Aspergillus nidulans (XprG) 490aa [pI 9.36; Mw 52839.41 Da-Expasy Compute pI/Mw tool]

```
.................18..........................46
MVTSFDPPPPAEPPADSSIDHNNKLLSFSMPVYPWTLLDYSFRRASISISAQLHGMFFLAESPWTTSPTE
NAPPQQGAELTCYRRNLFQITGSVTLPRALRYIITDTGDRIPIVAHELTVSATESVEGNSVKIISVPWKT
PAANDAGKDTGNSSNTAAKVEKEPPAIPLDMLTGQDLDADYATFPIAWKRLQFRVATANNGRRKELQQHF
VVRLRVVATLSTGMKTPICEVHSGPVIVRGRSPRNFQSRKDLPLSGSAAASRKNAQAAAASNNLTRTSPS
LTDKAKTVVKSSSPETSSNGVPQQSPPNWALATNSTLPPPTTTTLPHSSVYSQSSPEFSRPVEAHRRTTS
...................................................332
AIAAPINLSLLDDDSLNLSNGDSRPHTSFSNDLASKSLSVDSGRPVKMRKVSHSMPQAQSRSTSATFLNT
ANFQQMLPVPFTSESADVLYEYFPLGLEDWQGPVDAVYRPHVVHHTNMPQMKYITARGQSKRYFAAEDVF
```

Ser9 (in response to ionizing radiation in human p53): absent in *A. nidulans*
Ser15 (G1–S cell cycle checkpoint, apoptosis in human p53): absent in *A. nidulans*
Ser18 (glucose homeostasis in human p53): present in *A. nidulans*
Ser46 (apoptosis in human p53): present in *A. nidulans*
