# Supplementary material for: The Aspergillus nidulans ATM Kinase Regulates Mitochondrial Function, Glucose Uptake and the Carbon Starvation Response
Source: G3 (Bethesda). 2013 Nov 5;4(1):49–62. doi: 10.1534/g3.113.008607 (PMC3887539; doi:10.1534/g3.113.008607)
Supplement: Supporting Information [file supp_g3.113.008607_TableS1.pdf]

Table S1 Primers used in this study

Primers	Sequence
5' UTR XprG pRS426 fw	5'-GTAACGCCAGGGTTTTCCCAGTCACGACGGCACTACAATTCCCCAGCCCTT-3'
5' UTR XprG pyrG rv	5'-GTGCCTCCTCTCAGACAGAATGGCAAGGGTTCGGCTGTA-3'
pyrG alcA fw	5'-GCATTGTTTGAGGCGAATTCTGAAAAGCTGATTGTGATAGTTCC-3'
pyrG alcA fw	5'-GCATTGTTTGAGGCGAATTCTGAAAAGCTGATTGTGATAGTTCC-3'
alcA orf XprG fw	5'-AGTTAATTAGCGGTACCGGGATGGAGGGCTTCGACACC-3')
pMCB17 alcA rv primers	5'-CCCGGTACCGCTAATTAACT-3'
pRS426 orf XprG rv	5'-GCGGATAACAATTTCACACAGGAAACAGCTCAGACTGGGCGAGGTTCG-3'
TubC Fw	ATGCCGTCGCCGAAAG
TubC Rv	CATTCGGACGAGACATTC
XprG Fw	CGGCTGATCTGGAAAAGATTTCGC
XprG Rv	AAATGCTCCGCCTCAGCAAG

Tables S2-S4 are available for download as Excel files at http://www.g3journal.org/lookup/suppl/doi:10.1534/g3.113.008607/-/DC1.

Table S2 The list of *A. nidulans* genes significantly modulated upon carbon starvation (p<0.001) in either the wild-type, Δ*atmA* or both strains.

Table S3 Distribution of the genes in the subclusters of the hierarquical clustering.

Table S4 The overrepresented GO terms (Fisher's exact test, p<0.05) in the list of genes significantly modulated in the wild-type (WT) and Δ*atmA* strains in response to carbon starvation.
